# Temporal and Location Variations, and Link Categories for the Dissemination of COVID-19–Related Information on Twitter During the SARS-CoV-2 Outbreak in Europe: Infoveillance Study

**DOI:** 10.2196/19629

**Published:** 2020-08-28

**Authors:** Monika Pobiruchin, Richard Zowalla, Martin Wiesner

**Affiliations:** 1 GECKO Institute for Medicine, Informatics & Economics Heilbronn University Heilbronn Germany; 2 Consumer Health Informatics SIG German Association for Medical Informatics, Biometry & Epidemiology (GMDS e. V.) Cologne Germany; 3 Department of Medical Informatics Heilbronn University Heilbronn Germany; 4 Center for Machine Learning Heilbronn University Heilbronn Germany

**Keywords:** COVID-19, SARS-CoV-2, social media, public health, Twitter, infoveillance, infodemiology, infodemic, health informatics, disease surveillance

## Abstract

**Background:**

The spread of the 2019 novel coronavirus disease, COVID-19, across Asia and Europe sparked a significant increase in public interest and media coverage, including on social media platforms such as Twitter. In this context, the origin of information plays a central role in the dissemination of evidence-based information about the SARS-CoV-2 virus and COVID-19. On February 2, 2020, the World Health Organization (WHO) constituted a “massive infodemic” and argued that this situation “makes it hard for people to find trustworthy sources and reliable guidance when they need it.”

**Objective:**

This *infoveillance* study, conducted during the early phase of the COVID-19 pandemic, focuses on the social media platform Twitter. It allows monitoring of the dynamic pandemic situation on a global scale for different aspects and topics, languages, as well as regions and even whole countries. Of particular interest are temporal and geographical variations of COVID-19–related tweets, the situation in Europe, and the categories and origin of shared external resources.

**Methods:**

Twitter’s Streaming application programming interface was used to filter tweets based on 16 prevalent hashtags related to the COVID-19 outbreak. Each tweet’s text and corresponding metadata as well as the user’s profile information were extracted and stored into a database. Metadata included links to external resources. A link categorization scheme—introduced in a study by Chew and Eysenbach in 2009—was applied onto the top 250 shared resources to analyze the relative proportion for each category. Moreover, temporal variations of global tweet volumes were analyzed and a specific analysis was conducted for the European region.

**Results:**

Between February 9 and April 11, 2020, a total of 21,755,802 distinct tweets were collected, posted by 4,809,842 distinct Twitter accounts. The volume of #covid19-related tweets increased after the WHO announced the name of the new disease on February 11, 2020, and stabilized at the end of March at a high level. For the regional analysis, a higher tweet volume was observed in the vicinity of major European capitals or in densely populated areas. The most frequently shared resources originated from various social media platforms (ranks 1-7). The most prevalent category in the top 50 was “Mainstream or Local News.” For the category “Government or Public Health,” only two information sources were found in the top 50: US Centers for Disease Control and Prevention at rank 25 and the WHO at rank 27. The first occurrence of a prevalent scientific source was Nature (rank 116).

**Conclusions:**

The naming of the disease by the WHO was a major signal to address the public audience with public health response via social media platforms such as Twitter. Future studies should focus on the origin and trustworthiness of shared resources, as monitoring the spread of fake news during a pandemic situation is of particular importance. In addition, it would be beneficial to analyze and uncover bot networks spreading COVID-19–related misinformation.

## Introduction

### Overview

The emergence of SARS-CoV-2 and the associated COVID-19 [1] was first observed and described in China [2-6]. The subsequent spread across Asia [7] and Europe [8], including Northern Italy [9-12], in early 2020 sparked a significant increase in public interest and media coverage [13] including on the social media platforms Weibo [14] and Twitter [15,16]. During the following weeks, several SARS-CoV-2 infections were reported in other European countries [17,18] including the United Kingdom [19], Germany [20,21], France [22], and Spain [23].

According to Merchant and Lurie [24], several aspects play an important role in coping with the COVID-19 pandemic situation, especially in the digital age. First, “directing people to trusted sources” stands out, and neither a vaccine or drug against SARS-CoV-2 exists as of the time of writing. Second, the authors describe “social media as a diagnostic tool and referral system.” By monitoring related activities on different social media platforms, public authorities or research institutions can gather valuable insights into regional trends, country-specific trends, or even the global situation. Third, misinformation and rumors can quickly spread in a globally connected world [24,25]. Misbeliefs, fake news, and conspiracy theories pose a severe threat and might put people’s lives in danger [26]. In this context, Merchant and Lurie [24] propose a strategy of “counteracting misinformation” actively. In this way, they argue that “enabling a culture of preparedness” could be achieved.

In this context, the origin of information plays a central role in the dissemination of evidence-based information about the SARS-CoV-2 virus and the associated COVID-19. On February 2, 2020, the World Health Organization (WHO) constituted a “massive infodemic” and argued that this situation “makes it hard for people to find trustworthy sources and reliable guidance when they need it” [27,28].

Several trustworthy sources seem to be of particular interest [15,29]: research, public health, and government institutions, as well as news agencies or broadcasting companies and digital or print newspapers.

### Related Work

In 2009, Eysenbach [30] described the *infodemiology* and *infoveillance* concepts as a set of “public health informatics methods” to “analyze search, communication and publication behavior on the Internet.” During the 2009 H1N1 flu pandemic, Chew and Eysenbach [31] applied this concept for a content analysis of topic-related posts on Twitter in which they analyzed diseases-related trends, the origin of shared resources, and the sentiment expressed in swine flu tweets as posted via the platform.

Fu et al [32] analyzed how people reacted to the Zika epidemic in the Americas from 2015 to 2016. The authors analyzed 132,033 tweets with the keyword “zika” written in the languages English, Spanish, and Portuguese via the Twitter application programming interface (API). The authors reported that the top ranked shared resources originated from social media platforms such as “Facebook, Instagram, Twitter, YouTube, LinkedIn, Tumblr, the blogging site WordPress, [...] which accounted for 26% of all domains.” In the Zika study, the Centers for Disease Control and Prevention (CDC) and the WHO amounted to “0.06%” and “0.05%,” respectively. This corresponded to a 90th and 140th rank, respectively.

However, people do not only share evidence-based or trustworthy content in social media environments [33]. Southwell et al [34] pointed out that misinformation and perils exist that can lead to a spread of incorrect information, ambiguous meanings, and misperceptions, which can persist for a long period of time, and it can be resource intensive to counter misinformation “once it has enjoyed wide exposure.”

In the context of the current COVID-19 pandemic, an example of such incorrect information is the “5G conspiracy theory” [35], which led to phone masts being attacked in the United Kingdom [36].

Abd-Alrazaq et al [16] analyzed the content and sentiment of about 2.8 million COVID-19–related tweets retrieved via the Twitter standard search API written in the English language. They identified “four main themes: origin of the virus; its sources; its impact on people, countries, and the economy; and ways of mitigating the risk of infection” by applying topic modelling techniques using latent Dirichlet allocation. However, the analysis of shared resources and temporal and geographical variations of their 2.8 million tweets collection was not in the focus of their study.

### Aims of the Study

For this *infoveillance* study, during the early phase of the COVID-19 pandemic, the authors decided to focus on the social media platform Twitter, as the platform allows monitoring of the dynamic pandemic situation on a global scale in real time for different aspects of a topic, languages, as well as regions and even whole countries.

In this context, the research questions (RQs) of this study were as follows:

1. What tweet volume was observed among COVID-19–related hashtags at the beginning of the pandemic before and after the WHO announced the name of the disease?
2. How did information on COVID-19 and its associated impact spread during the epidemic situation in Europe from early February to early April 2020?
3. What proportion of information originates from public institutions, media channels, and scientific journals, and which channels stand out?

To the best of the authors’ knowledge, no similar COVID-19 study has been conducted on a comparable scale.

## Methods

### Study Design

This *infoveillance* study on the use of hashtags in the early onset of SARS-CoV-2 in the European countries consists of three stages. First, to answer RQ1, tweets with SARS-CoV-2– and COVID-19–related hashtags were collected. An analysis of which hashtags were used depending on 7-day intervals was conducted after the WHO announcement that named the disease on February 11, 2020. Second, based on the given geolocation information, the number of tweets from the European countries and their variations in time were analyzed (RQ2). Third, *European* tweets with online resources (ie, URL information) were extracted. The target of the URL was examined to determine its origin (eg, news agency, government institution, social media).

### Study Setting

In the early onset of the SARS-CoV-2 epidemic, several hashtags emerged worldwide. Based on the global Twitter trends and media coverage in January 2020, eight hashtags were initially included for collecting tweets beginning on February 9, 2020. In late February and at the beginning of March 2020, several other hashtags were increasingly used and, therefore, included in the study setting (see Multimedia Appendix 1). The special European focus was initiated by monitoring the worsening of the severe SARS-CoV-2 outbreak in the Northern Italian regions Lombardy and Emilia Romagna [10,11,37]. For this reason, the authors decided to add the two Italy-specific hashtags #coronavirusitaly and #coronavirusitalia that were prevalent around the third week of February 2020, as reported by Twitter trends at that time.

In total, 16 hashtags were selected for collecting COVID-19–related tweets for the purpose of temporal, geolocation, and link category analyses.

### Data Acquisition

#### Twitter Data

**Figure 1:**
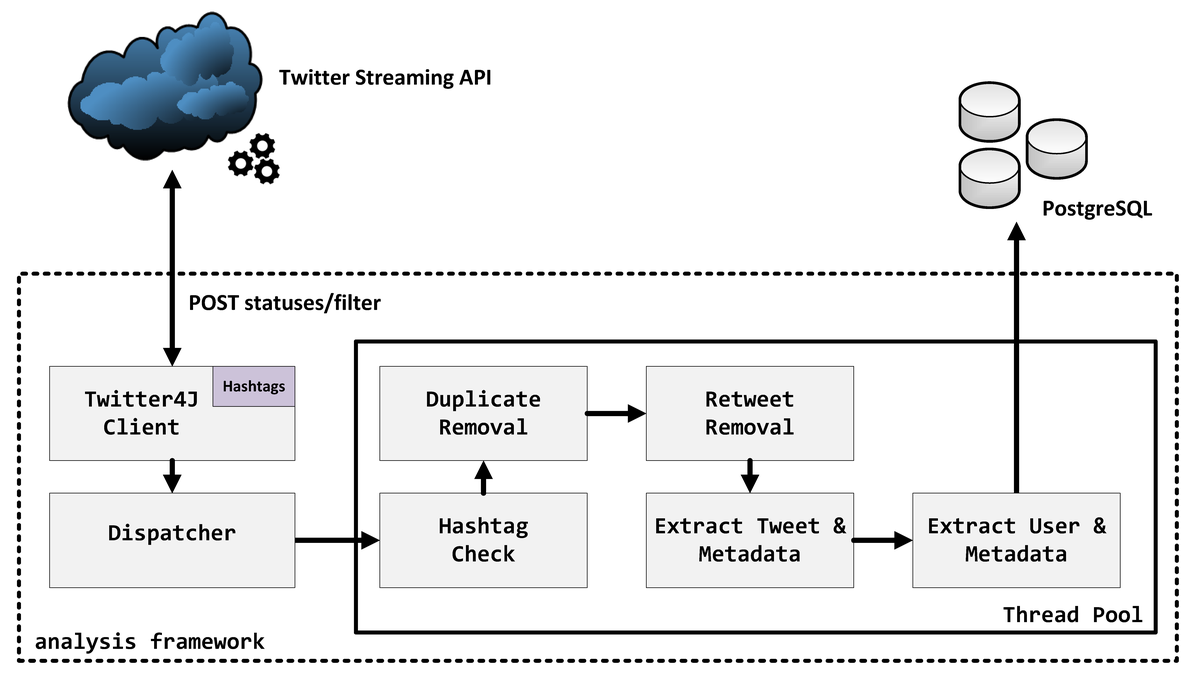
Workflow of the processing steps and involved software components: lines with arrows indicate processing workflow for each tweet *t* returned by the Twitter streaming API under the given hashtags included in this study. Each *t* was processed in parallel by the analysis framework to reach high-throughput processing for the large volume of COVID-19–related tweets. API: application programming interface.

For this study, tweets were collected between February 9 (midnight Central European Summer Time [CEST]) and April 11 (11:59 PM CEST), 2020, using the “Filter realtime Tweets” endpoint of the Twitter Streaming API [38] via the Java library *Twitter4J* [39] with the “standard” access level. To build the related filter query, the aforementioned hashtags (see Study Setting) were connected using the *OR* operator. Matching tweets were then processed by a self-implemented software framework written in Java (Oracle Corporation). Duplicate tweets as well as retweets were removed in this process. A tweet’s text, its metadata (eg, URLs appended to a tweet), as well as the user’s profile information were extracted. The results were then stored in a *PostgreSQL* in v10.12 [40]. The processing workflow is depicted in Figure 1.

### Twitter Analysis

In the context of this study, a tweet contains at least one of the 16 hashtags ([a] to [p] in Multimedia Appendix 1) as described in the section Study Setting. All analyses were conducted based on these hashtags. Additionally, detected hashtags mentioned in a specific tweet were not considered.

Twitter provides geographic information of a Twitter user’s location (ie, latitude φ and longitude λ) [41]. According to the Twitter API, such geographic information can either be an exact point location or a bounding box (ie, a larger area or an entire region). Given such a bounding box, our analysis framework computed the geometric center of it and used this information as a point location.

However, Twitter users can deactivate sharing of their location information. If the geographic information was given, we leveraged this information to plot tweet locations on a map. The authors defined the European area with geographical limits ranging from 34.839°<*φ*<75.00° latitude (excluding the islands of Svalbard) and –31.26192°<λ<59.34569° longitude. Corresponding maps visualize the geographical and temporal spread of the pandemic via tweets in the European countries.

### Link Category Analysis

Twitter users can share external resources to disseminate important information or to support an individual statement. In the context of RQ3, all URLs shared by users, excluding retweets or citations, were of particular interest. Before categorization could be conducted, URLs shortened by a corresponding service (eg, bit.ly, buff.ly) were resolved in an automatic procedure using the *crawler4j* framework [42]. In case shortened links could not be resolved, those URLs were left as originally captured via our analysis framework (see Figure 1). Next, domain aggregation was applied on each unique URL [43] (ie, “https://mhealth.jmir.org/” becomes “jmir.org”). The domain aggregation was conducted by a self-implemented software written in Java using the public suffix list provided by the Mozilla Foundation [44]. This transformation was conducted on a Ubuntu 18.04 LTS 64-bit computer running Java 11.0.7 on April 22, 2020.

The most prevalent (n=250) domain-aggregated URLs associated with a web site, as shared by Twitter users, were categorized according to the categories introduced by Chew and Eysenbach [31] in 2010. The category “No Reference” was not considered, as only tweets containing at least one URL were included in the link category analysis. Two additional categories with respect to RQ3 were introduced: (1) “Scientific resource” (eg, journal, magazine, preprint servers, or university provided COVID-19 dashboard) and (2) “URL Shortener.”

The categorization was conducted manually by all three authors independently. Subsequently, the interrater reliability metrics percent agreement (PA) [45] and Fleiss κ [46] were computed. If there was a split situation, the authors discussed the specific case and resolved all unclear cases.

### Statistical Analysis

Data were analyzed with the statistics software *R* (The R Foundation for Statistical Computing) in version 3.6.3 (February 29, 2020) on a Ubuntu 18.04 LTS 64-bit computer. The *R* package *ggplot2* [47] was used for visualization of tweets’ and hashtags’ temporal and geographic variations. In addition, *R* was used to compute PA and Fleiss κ.

### Ethical Approval

This article does not contain any study of human participants performed by any of the authors. For this reason, no formal ethical approval is required.

## Results

### Principal Findings

Since the emergence of the first reports of human SARS-CoV-2 virus infections in China in late December 2019 and early January 2020 [5], the public interest and social media use grew steadily. The volume of #covid19-related tweets increased with the WHO announcement after February 11 [1] and stabilized at the end of March at a high level. Several hashtags were used in the early phase of the SARS-CoV-2 outbreak, such as #nCov2019, #nCov19, #nCov, and #2019nCov. Those earlier forms of referencing COVID-19 did not show substantial volume after the WHO announcement. Thus, the naming of the disease was a major signal to address the public audience with a public health response via social media platforms (ie, Twitter).

The situation in Europe changed with the #coronavirus outbreak in (Northern) Italy [37]. Public interest rose with climbing numbers of infections, as Italy became a hot spot of the epidemiological situation on the European continent. Country-specific hashtags were used to report on the Italian situation, and with the spread of the disease in Europe, users in other countries engaged with their individual hashtags such as #coronavirusES (Spain), #coronaFrance (France), and #coronavirusDeutschland (Germany). Nevertheless, neutral hashtags such as #covid19 or #COVID—19 showed constant use and corresponding high volume.

Many Twitter users expressed their engagement by sharing either image or multimedia content, or URLs to external references of which they believed provided important information to other users. A quarter of the observed tweets were posted with images, and another third provided links to external references. Of these references, 1 out of 5 cross-linked to posts in social media platforms (eg, YouTube, Instagram, or Reddit). Mainstream or local news resources were shared by 1 out of 8 posts. References to information provided by governmental or public health institutions and COVID-19–related scientific resources were posted rarely (1 out of 100).

### Sample Characteristics

Between February 9 (midnight CEST) and April 11 (11:59 PM CEST), 2020, a total of 21,755,802 distinct tweets posted under the 16 hashtags were collected and stored in the study database. Those tweets were posted by 4,809,842 distinct Twitter accounts of which 83,560 were verified by the platform itself [48]. On average, each tweet contained 3.18 hashtags (min=1, max=47). The most prevalent languages were identified according to Twitter’s language classification [49] and are listed in Table 1.

Of the 21,755,802 tweets, 25.78% (n=5,608,189) of tweets used (animated) images. Likewise, 4.95% (n=1,076,180) of all posts shared multimedia material (ie, videos). In total, 7,753,841 (34.16%) posts shared external resources. On average, a tweet referencing an external URL contained 1.04 URLs (min=1, max=10).

**Table 1:** Language distribution of the study sample.

Rank	Language	Observations (n=21,755,802), n (%)
1	English	11,829,991 (54.38)
2	Spanish	3,037,910 (13.96)
3	Undefined	1,325,729 (6.09)
4	French	1,246,211 (5.73)
5	Italian	898,979 (4.13)
6	Turkish	493,155 (2.27)
7	German	446,502 (2.05)
8	Portuguese	310,332 (1.43)
9	Indonesian	242,068 (1.11)
10	Hindi	228,966 (1.05)
11	Thai	227,665 (1.05)
12	Japanese	220,032 (1.01)
13	Arabic	195,541 (0.90)
14	Dutch	178,768 (0.82)
15	Catalan	155,535 (0.71)
≥16	Other	718,418 (3.30)

### Twitter Analysis

#### Temporal Variations of Tweets

The total number of occurrences for each hashtag is presented in Table 2. The data shows a heterogeneous distribution of hashtag volume. The hashtags #WuhanVirus and #Wuhan were less frequently used than more *generic* hashtags such as #covid19. The four top hashtags in the study database represent 93.98% (24,203,025/25,754,619) of all hashtags mentioned: (1) #coronavirus, (2) #covid19, (3) #COVID—19, and (4) #Covid_19.

**Figure 2:**
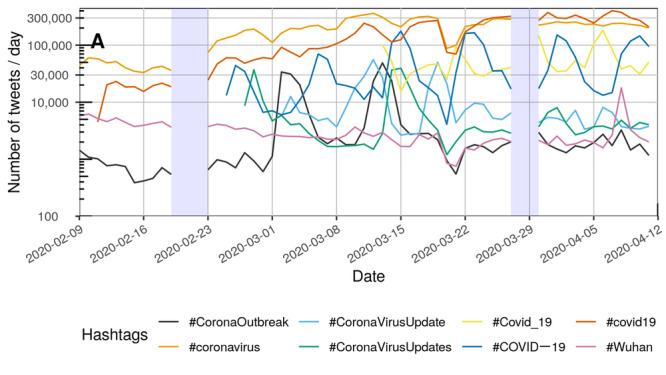
Number of tweets per day for the hashtags ranked 1-8 (see Table 2) between February 9, 2020, and April 11, 2020, on a logarithmic scale. The capital letter "A" represents the naming of the disease by the World Health Organization on February 11, 2020. Blue rectangles: No tweets were collected between February 20 and 22 as well as between March 28 and 29 due to technical issues.

**Figure 3:**
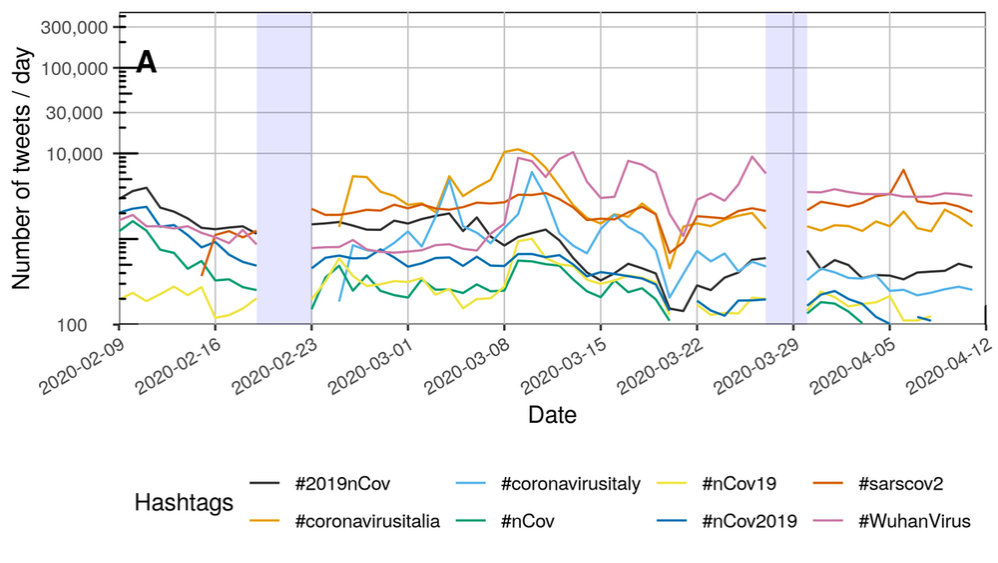
Number of tweets per day for the hashtags ranked 9-16 (see Table 2) between February 9, 2020, and April 11, 2020, on a logarithmic scale. The capital letter "A" represents the naming of the disease by the World Health Organization on February 11, 2020. Blue rectangles: No tweets were collected between February 20 and 22 as well as between March 28 and 29 due to technical issues.

Figures 2 and 3 each depict the number of tweets per day that contained at least one of the COVID-19–related hashtags.

Overall, the number of daily tweets rose during the study period. The use of #covid19 increased throughout February and March. The trend was similar to the use of #coronavirus. However, the use of the hashtag #COVID—19 was fluctuating periodically. Similar peaks in usage could be detected for #Covid_19. Multimedia Appendix 2 provides a complete list of all depictions of temporal variations for each hashtag separately.

**Table 2:** Number of tweets per hashtag in ranked order within 7-day intervals.

Rank	Hashtag	Feb 9, n (%)	Feb 16^a^, n (%)	Feb 23, n (%)	Mar 1, n (%)	Mar 8, n (%)	Mar 15, n (%)	Mar 22^b^, n (%)	Mar 29^b^, n (%)	Apr 5, n (%)	Total, n (%)
1	#coronavirus	337,478 (65.91)	159,013 (57.32)	1,000,498 (61.89)	1,287,233 (56.53)	2,057,022 (51.46)	1,609,870 (42.35)	1,537,502 (38.20)	1,553,761 (34.30)	1,587,109 (32.42)	11,129,486 (42.92)
2	#covid19	84,892 (16.58)	79,568 (28.68)	354,548 (21.93)	553,564 (24.31)	1,114,486 (27.88)	1,263,151 (33.23)	1,599,310 (39.73)	1,972,964 (43.55)	2,130,548 (43.52)	9,153,031 (35.30)
3	#COVID—19	—^c^	—	113,479 (7.02)	177,572 (7.80)	209,752 (5.25)	354,910 (9.34)	515,987 (12.82)	415,712 (9.18)	472,185 (9.65)	2,259,597 (8.71)
4	#Covid_19	—	—	—	—	152,901 (3.83)	297,663 (7.83)	230,274 (5.72)	443,837 (9.80)	536,257 (10.95)	1,660,932 (6.40)
5	#CoronaVirusUpdate	—	—	—	40,835 (1.79)	144,616 (3.62)	102,272 (2.69)	42,775 (1.06)	31,607 (0.70)	32,773 (0.67)	394,878 (1.52)
6	#CoronaVirusUpdates	—	—	56,143 (3.47)	25,440 (1.12)	47,877 (1.20)	76,200 (2.00)	19,024 (0.47)	29,460 (0.65)	27,665 (0.57)	281,809 (1.09)
7	#CoronaOutbreak	6237 (1.22)	2305 (0.83)	6070 (0.38)	96,691 (4.25)	105,210 (2.63)	16,081 (0.42)	10,052 (0.25)	11,611 (0.26)	14,131 (0.29)	268,388 (1.03)
8	#Wuhan	35,142 (6.86)	17,230 (6.21)	24,242 (1.50)	17,421 (0.77)	19,081 (0.48)	12,770 (0.34)	11,467 (0.28)	13,648 (0.30)	31,707 (0.65)	182,708 (0.70)
9	#WuhanVirus	10,301 (2.01)	4301 (1.55)	5522 (0.34)	5825 (0.26)	47,418 (1.19)	30,699 (0.81)	28,420 (0.71)	22,942 (0.51)	22,690 (0.46)	178,118 (0.69)
10	#coronavirusitalia	—	—	18,838 (1.17)	24,697 (1.08)	46,517 (1.16)	11,694 (0.31)	9828 (0.24)	9167 (0.20)	11,499 (0.23)	132,240 (0.51)
11	#sarscov2	369 (0.07)	4881 (1.76)	14,964 (0.93)	16,913 (0.74)	19,615 (0.49)	11,400 (0.30)	11,915 (0.30)	16,710 (0.37)	22,215 (0.45)	118,982 (0.46)
12	#2019nCov	18,041 (3.52)	5496 (1.98)	10,226 (0.63)	11,172 (0.49)	6330 (0.16)	2389 (0.06)	2471 (0.06)	3167 (0.07)	2939 (0.06)	62,231 (0.24)
13	#coronavirusitaly	—	—	3379 (0.21)	12,238 (0.54)	15,137 (0.38)	7082 (0.19)	3391 (0.08)	2562 (0.06)	1750 (0.04)	45,539 (0.18)
14	#nCov2019	11,426 (2.23)	2718 (0.98)	4255 (0.26)	3799 (0.17)	3951 (0.10)	2053 (0.05)	1043 (0.03)	1206 (0.03)	727 (0.01)	31,178 (0.12)
15	#nCov	6536 (1.28)	1265 (0.46)	2086 (0.13)	1831 (0.08)	2935 (0.07)	1385 (0.04)	565 (0.01)	856 (0.02)	535 (0.01)	17,994 (0.07)
16	#nCov19	1618 (0.32)	645 (0.23)	2382 (0.15)	1707 (0.07)	4151 (0.10)	1893 (0.05)	979 (0.02)	1166 (0.03)	768 (0.02)	15,309 (0.06)
Total	N/A^d^	512,040 (1.97)	277,422 (1.07)	1,616,632 (6.23)	2,276,938 (8.78)	3,996,999 (15.41)	3,801,512 (14.66)	4,025,003 (15.52)	4,530,376 (17.47)	4,895,498 (18.88)	25,932,420 (100.00)

^a^During this 7-day interval technical issues occurred for approximately 3 days.

^b^During this 7-day interval technical issues occurred for approximately 1 day.

^c^No data available.

^d^N/A: not applicable.

#### Geographical Variations of Tweets

**Figure 4:**
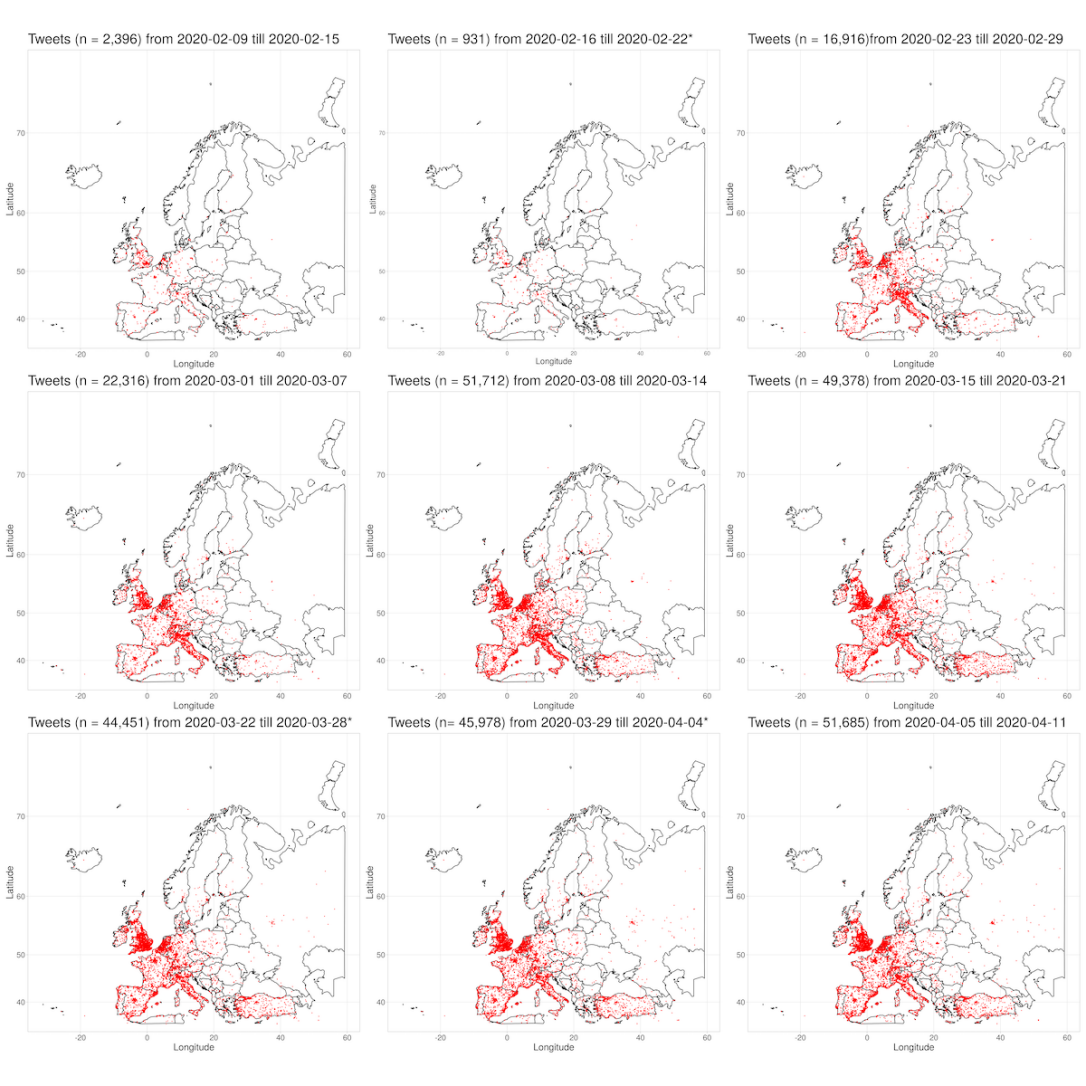
Geolocation information of COVID-19–related tweets depicted for each 7-day interval. From top left (February 9, 2020, to February 15, 2020) to bottom right (April 5, 2020, to April 11, 2020). A single red dot denotes one tweet. Tweets with the same geographical information are plotted on top of each other. *No tweets were collected between February 20, 2020, and February 22, 2020, and between March 28, 2020, and March 29, 2020, due to technical issues.

In the beginning of February 2020, the SARS-CoV-2 epidemic spread over Europe. The northern regions of Italy especially had a massive outbreak of COVID-19 [9]. To investigate the tweets’ volume spread, all tweets that contained geographic coordinates were included in this subanalysis. Longitude and latitude information were available for 4.40% (957,947/21,755,802) of the tweets in the study database; filtered for the longitude and latitude representing the geographical borders of Europe, 29.83% (285,763/957,947) of tweets qualified. Each tweet was plotted in a geographical map of Europe for each 7-day interval in the observation period (see Figure 4).

**Figure 5:**
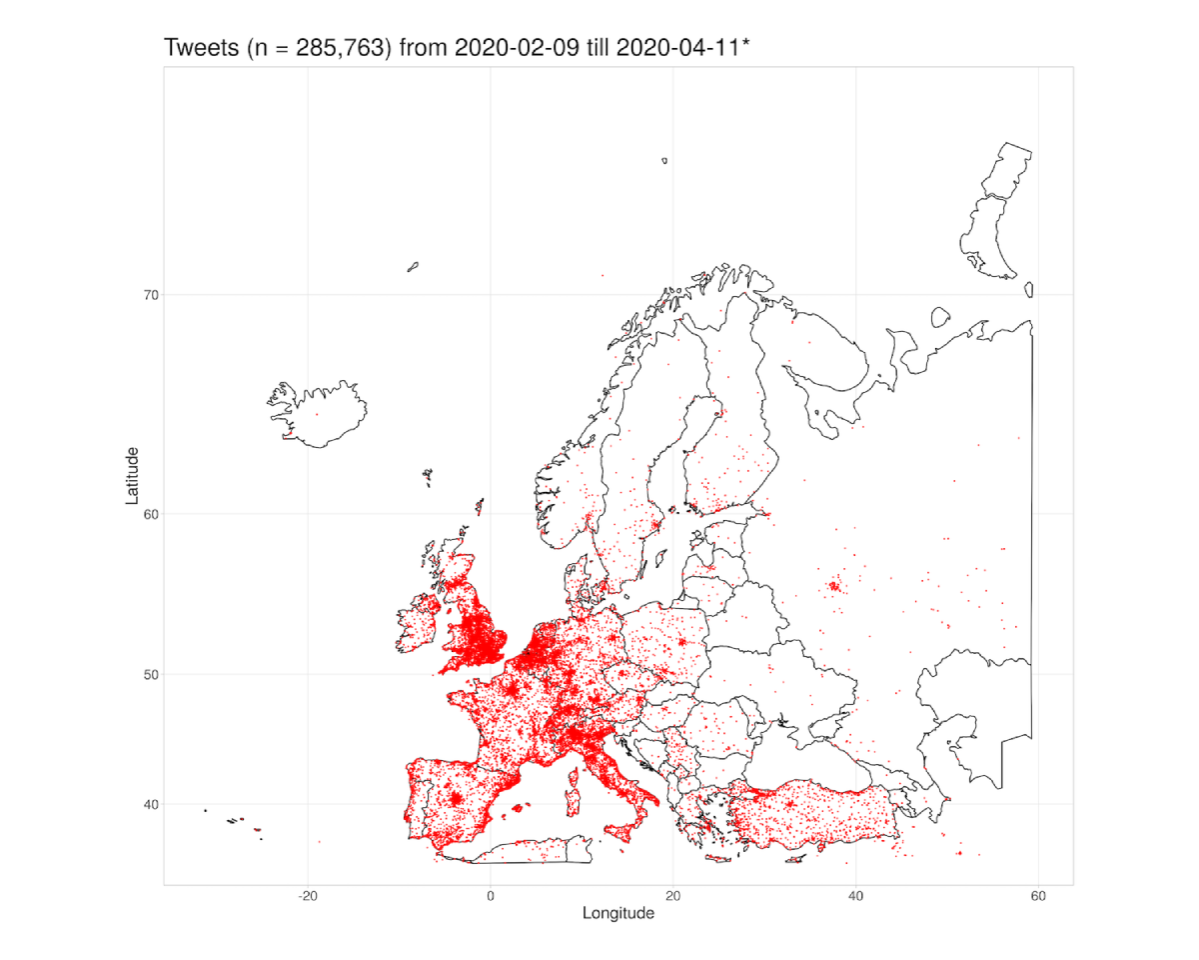
Cumulative depiction of all tweets in European countries between February 9 and April 11, 2020. Each red dot denotes one tweet. Tweets with the same geographical information are plotted on top of each other. *No tweets were collected between February 20, 2020, and February 22, 2020, and between March 28, 2020, and March 29, 2020, due to technical issues.

For an animated video that covers the observation period between February 9, 2020, and April 11, 2020, see Multimedia Appendix 3. For a high-resolution collection of the subplots in Figure 4, see Multimedia Appendix 4. In addition, Figure 5 presents a cumulative plot of all 285,763 tweets that provided geolocation information for the European continent. More tweets could be observed in the vicinity of countries’ capitals (eg, Paris, Madrid, Vienna, or Berlin) or in densely populated areas such as the Benelux Union or South England. A higher number of tweets with geolocations was observed in Central and Western European countries than compared to Eastern Europe. Interestingly, tweet volumes in Turkey seemed to be higher than in surrounding countries.

### Link Category Analysis

The most prevalent (n=250) domain-aggregated URLs were categorized by three researchers independently according to the categories introduced by Chew and Eysenbach [31]. These URLs accounted for 46.38% (3,596,538/7,753,841) of all shared resources in our study database. The three researchers achieved a PA of 0.628 and a Fleiss κ of 0.639. According to Landis and Koch [50], these κ values correspond to a “substantial agreement.” In 4.4% (11/250) of the cases, no majority vote was achieved and those were subsequently cleared by discussion among all of the authors. The link category “Not Accessible” was not selected, as all domains were accessible by every researcher.

Table 3 presents the top 50 shared domains and their link categories, and the occurrences of each domain in the study database. The complete list of the top 250 can be found in Multimedia Appendix 5. The most frequently shared resources originated from various social media platforms and are represented in the ranks 1-7. Cross-linking resources on social media (even on the same platform [ie, Twitter]) could be observed. The most prevalent category in the top 50 was “Mainstream or Local News.” The resources of the newspapers The Guardian and the New York Times were the leading domains in this category, followed by the broadcasting services CNN and BBC. Only two domains qualified for the category “Government or Public Health” in the top 50: CDC at rank 25 and the WHO at rank 27. No scientific resource was contained in the top 50. The first occurrence was the British journal Nature at rank 116.

**Figure 6:**
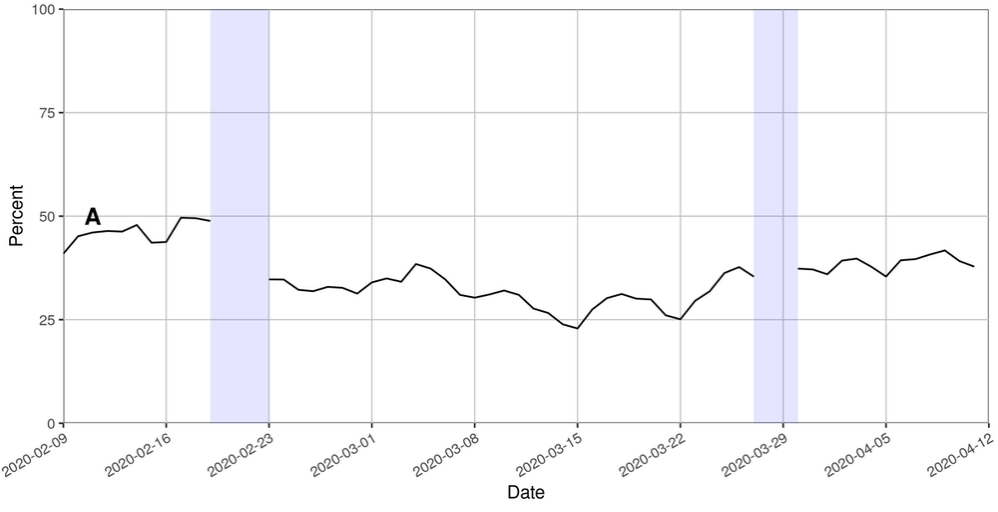
The relative proportion of tweets with links to external resources. The capital letter "A" represents the naming of the disease by the World Health Organization on February 11, 2020. Blue rectangles: No tweets were collected between February 20 and 22 as well as between March 28 and 29 due to technical issues.

The relative proportion of tweets that shared references to external resources varied during the study period. A longitudinal subanalysis revealed a constant trend without major peaks for each day of the study (see Figure 6).

**Table 3:** Categorized top 50 shared website domains. Total number of occurrences of external references (n=7,753,841).

Link category and rank			Domain		Occurrences, n (%)
**Mainstream or local news**					
	8	theguardian.com		52,733 (0.68)	
	11	nytimes.com		42,735 (0.55)	
	13	cnn.com		35,494 (0.46)	
	14	bbc.co.uk		28,286 (0.36)	
	15	washingtonpost.com		27,316 (0.35)	
	19	bbc.com		25,853 (0.33)	
	28	nyti.ms		17,720 (0.23)	
	30	reuters.com		17,589 (0.23)	
	33	cnbc.com		17,321 (0.22)	
	35	bloomberg.com		16,330 (0.21)	
	37	elpais.com		15,888 (0.20)	
	38	ouest-france.fr		14,976 (0.19)	
	40	francetvinfo.fr		14,609 (0.19)	
	41	scmp.com		14,072 (0.18)	
	43	reut.rs		13,637 (0.18)	
	46	forbes.com		13,242 (0.17)	
	48	nypost.com		12,464 (0.16)	
	49	businessinsider.com		12,433 (0.16)	
**News blog, feed, or niche news**					
	17	medium.com		26,201 (0.34)	
	42	zazoom.it		13,926 (0.18)	
	45	zazoom.info		13,303 (0.17)	
	47	topicza.com		12,960 (0.17)	
**Government or public health**					
	25	cdc.gov		19,729 (0.25)	
	27	who.int		18,298 (0.24)	
**Personal blog**					
	23	wordpress.com		22,376 (0.29)	
**Social network**					
	1	twitter.com		378,508 (4.88)	
	2	youtu.be		365,716 (4.72)	
	3	instagram.com		290,336 (3.74)	
	5	youtube.com		144,502 (1.86)	
	6	facebook.com		95,166 (1.23)	
	7	linkedin.com		79,787 (1.03)	
	16	pscp.tv		26,823 (0.35)	
**Online store**					
	20	amzn.to		25,378 (0.33)	
**Scientific resource^a^**					
	—^b^	—		—	
**URL shortener^a^**					
	10	tinyurl.com		44,768 (0.58)	
	24	trib.al		21,409 (0.28)	
**Other**					
	4	paper.li		204,077 (2.63)	
	9	google.com		47,184 (0.61)	
	12	chng.it		41,316 (0.53)	
	18	fiverr.com		25,905 (0.33)	
	21	ift.tt		23,304 (0.30)	
	22	avaaz.org		22,938 (0.30)	
	26	arcgis.com		18,604 (0.24)	
	29	worldometers.info		17,670 (0.23)	
	31	yahoo.com		17,588 (0.23)	
	32	apple.news		17,584 (0.23)	
	34	openstream.co		16,442 (0.21)	
	36	goo.gl		16,310 (0.21)	
	39	joinzoe.com		14,827 (0.19)	
	44	shoutcast.com		13,635 (0.18)	
	50	dy.si		12,343 (0.16)	

^a^Link category as extension of the list given in Chew and Eysenbach [31].

^b^No domain qualified for a rank below or equal to 50. The full listing with all scientific resources under this category is found in Multimedia Appendix 5.

## Discussion

### Principal Results

The COVID-19–related tweet volume observed in this study increased constantly during the weeks of February 2020. However, this study did not investigate whether tweet volumes correlated with infection or death rates in different European countries. It can be assumed that increasing SARS-CoV-2 infection figures correlate with increased public interest and engagement on social media platforms. In addition to rising infection rates, several other factors such as the death of a celebrity due to COVID-19 could have increased public interest in the progress of the pandemic. In this context, the hashtag-specific analysis revealed that #COVID—19 and #Covid_19 in particular were fluctuating periodically without a clear connection to specific events.

The analysis over time revealed that the first Twitter *hot spots* in Europe developed not only in the capital cities of London and Paris but also in the region of Milan, Italy. The northern regions of Italy showed a sharp increase in tweet volume in the beginning and middle of February.

As the epidemic spread further over Europe, an increase of tweet volume over most of Western and Central Europe could be observed. However, the increase of tweets was not that prevalent in Eastern European countries (eg, Czech Republic, Poland, Romania) and in Southeastern Europe (eg, Serbia, Croatia). The public in Turkey increased their Twitter activity around the second week of March (see Figure 4) when the first COVID-19 case was officially confirmed by the Turkish health authorities.

The most frequently shared resources linked to various social media platforms and were represented by the ranks 1-7. The CDC website reached the 25th rank and the WHO website the 27th rank in the top 250 shared domain analysis. By contrast, the first occurrence of a prevalent scientific source is Nature at rank 116. Nevertheless, it was surprising that a high-class journal such as Nature was only directly referenced in 0.08% (6043/7,753,841) of the links to external resources shared on Twitter. Likewise, this finding applied for other scientific sources: Science (rank 147; 4615/7,753,841, 0.06%), The New England Journal of Medicine (rank 154; 4405/7,753,841, 0.06%), medRxiv (rank 170; 4123/7,753,841, 0.05%), and Johns Hopkins University (rank 199; 3586/7,753,841, 0.05%). Even with these numbers at hand, it remains an open question whether direct references to scientific sources should be included more actively for the purpose of public health communication on Twitter or not, given that a broad media coverage, which translates scientific language for a broader audience, seems necessary to disseminate important COVID-19–related research results to the public.

### Limitations

Many social media platforms were used to share personal opinions, information, and news or stories around a particular topic. In the context of the COVID-19 pandemic, different platforms were in the public interest. In the setting of this study, only contributions on the platform Twitter were investigated and public disease-related data was analyzed. For this reason, the reported findings may not be mapped and applicable to other social networks such as Facebook, Reddit, or YouTube.

Amplification of particular tweets can increase visibility of certain resources shared by users. In this context, retrospective queries and related analyses were limited by the capabilities as given by the “Post, retrieve, and engage with Tweets” endpoint [51] of the Twitter API with the “standard” access level. For this reason, the authors could not update the data collection of tweets at the end of the study period regarding retweets and likes. Consequently, no deep analysis of certain resources’ popularity could be conducted. It remains a future task to analyze these relationships, even though it seems impractical given the “standard” access level.

In addition, the Twitter API ensures, that privacy of nonpublic tweets is respected. This is why the “Filter realtime Tweets” endpoint [38] does not return privately posted tweets. Therefore, those users and tweets could not be included in this study. Yet, it is estimated that only a small proportion of Twitter users configure their account as fully private.

Most Twitter users configure their individual privacy settings to hide their personal geolocation. For this reason, the analyses of geographical variations was limited to a comparatively low amount of data. In the context of our study, geolocation data was only available for 4.40% (957,947/21,755,802) of the collected tweets. However, this subsample still accounts for around 1 million tweets in total. In this context, the study found Eastern European users of Twitter to be less engaged during the study period. This might originate from low Twitter adoption rates in Eastern Europe [52].

This study investigated the SARS-CoV-2 outbreak situation in Europe with a specific interest. This originated from the epidemic spread of the virus in Europe, starting in Italy [9-11]. This spread was accompanied by increased media coverage and public interest in Europe [21,53,54] and worldwide [55]. Researchers of different disciplines started analyzing regional differences among European countries such as Italy, Spain, France, Germany, or Austria [17]. In the context of this study, this motivated RQ2 and the specific analyses as reported. It is worth emphasizing, however, that the European languages cannot be mapped easily to very fine-grained country borders on classical maps. Therefore, the European region had to be approximated by using the geo-information and the bounding box, as described in the Methods section, defined by corresponding geo-coordinates. This resulted in a subsample of 285,763 tweets for the European region subanalysis.

Furthermore, the special European focus was initiated by monitoring the worsening of the severe SARS-CoV-2 outbreak in the Northern Italy regions of Lombardy and Emilia Romagna [11,37]. For this reason, the authors decided to add two Italy-specific hashtags that were prevalent around the third week of February 2020, as reported by Twitter trends at that time. However, it should be noted that those two hashtags account for only 0.18% (#coronovirusitaly; 45,439/25,932,420) and 0.51% (#coronavirusitalia; 132,240/25,932,420) of all hashtag usages in the study’s data collection. As the spread of the virus progressed over several countries in Europe, many other country-specific hashtags appeared in Twitter trend statistics. The authors decided to avoid including all possible variations and country-specific subhashtags. This possibility limits comparisons among different countries in Europe. Nevertheless, the generic hashtags for COVID-19 remained stable over the full study period. Thus, tweets can be found in the data collection for every European country.

The collection of data was conducted in real time. Sadly, due to technical issues on February 20-22 and March 28 and 29, 2020, data could not be collected during these time spans. The issue in February originated from a loss of connectivity to the *PostgreSQL* study database, which was not discovered for around 48 hours during a weekend. A second, technical issue in late March resulted from an unexpected memory allocation problem on the processing server. Once the issue was resolved by a software patch, the system was capable of collecting and storing tweets correctly again.

### Comparison With Prior Work

During the 2009 H1N1 flu pandemic, Chew and Eysenbach [31] applied the *infoveillance* concept for a content analysis for which they “archived over 2 million Twitter posts containing keywords ‘swine flu’, ‘swineflu’ and/or ‘H1N1.’” The authors analyzed diseases-related trends, the origin of shared resources, and the sentiment expressed in swine flu tweets. In our study, more than 20 million COVID-19–related tweets were analyzed for temporal or geographical characteristics and trends as well as for the link category of external resources. In the 2009 study [31], the authors found that “government and health agencies were only linked 1.5% of the time.” For a top 250 list, this low proportion is confirmed by our findings (78,786/7,753,841, 1.02%). Chew and Eysenbach [31] found that “news websites were the most popular sources (23.2%).” Likewise, our analysis revealed that the link category “Mainstream and local news” was represented by 11.97% (928,467/7,753,841), which was substantially lower than in 2009. In this context, our findings suggest that Twitter users cross-reference to Twitter itself or to other social media platforms (1,406,419/7,753,841, 18.14%), whereas this group was reported to represent only 2% of the corresponding category in the study by Chew and Eysenbach [31]. Moreover, the authors of the H1N1 flu study reported that “61.8% of all tweets had links [..].” In our study, this proportion was found to be 34.16% (7,431,226/21,755,802), which was substantially lower.

Fu et al [32] analyzed how people reacted to the Zika epidemic in the Americas from 2015 to 2016. The authors analyzed 132,033 tweets with the key word “zika” written in the languages English, Spanish, and Portuguese via the Twitter API. The authors reported, that the top ranked shared resources originated from social media platforms such as “Facebook, Instagram, Twitter, YouTube, LinkedIn, Tumblr, the blogging site WordPress, [..] which accounted for 26% of all domains.” This could be confirmed by our results, as social media platforms were ranked on the positions 1-7 accounting for 18.14% (1,406,419/7,753,841) of all shared resources. In the Zika study, the CDC and the WHO accounted for 0.06% and 0.05%, respectively. This corresponded to a 90th and 140th rank, respectively, compared to a 25th (19,729/7,753,841, 0.25%) and a 27th (18,298/7,753,841, 0.24%) rank, respectively, in our analysis on shared resources. The comparison suggests that public health-related material provided via the CDC or the WHO was shared more frequently than during the Zika outbreak between 2015 and 2016. This increase might originate from multiple reasons: improved, timely provisioning of disease-related material by either the CDC, the WHO, or both; higher awareness of the public for quality aspects of material and evidence-based sources; or the use of easy language or easily comprehendible infographics by the public health teams of the CDC, the WHO, or both.

Abd-Alrazaq et al [16] analyzed the content and sentiment of about 2.8 million COVID-19–related tweets, retrieved via the Twitter standard search API, written in the English language*.* By contrast, our study design made use of Twitter’s real-time Streaming API, which allows for a constant intake to the study database. In [16], the authors made use of the search terms “corona,” “2019-nCov,” and “COVID-19.” In our study, we monitored 16 hashtags for a time span of 9 weeks. This resulted in a data collection with a total of approximately 21.8 million topic-related tweets. With our analysis framework, we were able to monitor specific regions (Europe) and countries, in particular the SARS-CoV-2 outbreak in Italy.

### Future Directions

This study demonstrates how COVID-19–related tweets can be analyzed for a certain region (Europe). With the continuous progression of the pandemic situation, which is to be expected in the next months worldwide, further regions should be analyzed in-depth. Therefore, the authors encourage other researchers to contribute their analyses with a special focus on regions such as Africa, South and North America, or Asia. Moreover, different analysis techniques can be leveraged to learn more about what users share in the current pandemic situation. For this purpose, one could use sentiment analysis or conduct social network graph analysis to uncover patterns that might be hidden in the data. Sentiment analysis is of particular interest, as it could reveal differences between regions or even between several countries, such as demonstrated by Abd-Alrazaq et al [16] for tweets written in the English language [16].

Long-term Twitter monitoring based on geographical data could be a supporting tool for local health authorities. With an average tweet volume per city, region, or even country, significant peaks well above the 7-day average could be reported to official institutions quickly in an electronic, interoperable format. In this sense, an automated analysis tool could be an extension of our software components to capture pandemic-related tweets in real time.

Future studies should also focus on the origin and trustworthiness of shared resources. Monitoring the spread of fake news during a pandemic situation seems of particular importance [24,26]. Timely measures to fight and reduce the spread of COVID-19 misinformation could thus be supported. In addition, it would be beneficial to analyze and uncover bot networks spreading COVID-19–related misinformation. In this study, we could uncover periodicity of at least one hashtag (#COVID—19). This might be linked to a hidden bot network, which justifies further investigation.

In future work, the authors intend to publish the data collection according to the Developer Agreement and Policy of Twitter [56]. Other researchers might analyze this data collection with a different focus or with their own scientific perspective. By providing this data set, the requirement of providing one’s own technical infrastructure would pose no barrier for non–computer science disciplines. We hope to provide this data set publicly, regularly updated in 1 week intervals.

### Conclusions

The naming of the disease by the WHO on February 11, 2020 [1], was a major signal to address the public audience with a public health response via social media platforms. The volume of #covid19-related tweets increased after the WHO announcement and stabilized at the end of March at a high level.

During the spread of the SARS-CoV-2 virus in Europe between February 2020 and early April 2020, the public interest and media coverage increased rapidly. Consequently, the engagement of citizens on social media platforms rose accordingly. On April 16, 2020, Dr Hans Kluge, the WHO director for Europe, characterized the situation as “we remain in the eye of the [COVID-19] storm” [57]. The findings of this study allow for a detailed analysis for the European region and how citizens of different European countries shared their opinions, experiences, and concerns on Twitter. The detailed maps of Europe are available for each 7-day interval starting on February 9, 2020.

Social media platforms were ranked at the position of 1-7, counting for 18.14% (1,406,419/7,753,841) of all shared resources. The CDC website reached the 25th rank (19,729/7,753,841, 0.25%) and the WHO website the 27th rank (18,298/7,753,841, 0.24%) of the top 250 shared domain analysis. Future studies should focus on the origin and trustworthiness of shared resources, as monitoring the spread of fake news during a pandemic situation is of particular importance.

## Appendix

Multimedia Appendix 1 Listing of all hashtags included in the study.

Multimedia Appendix 2 Temporal variations of hashtag frequencies between February 9, 2020, and April 11, 2020.

Multimedia Appendix 3 Animated video of the geographical variation of tweets in the European countries.

Multimedia Appendix 4 Seven-day interval plots of the geographical variation of tweets in all European countries.

Multimedia Appendix 5 Categorized top 250 shared (web site) domains.

## Abbreviations

API: application programming interface
CDC: Centers for Disease Control and Prevention
CEST: Central European Summer Time
PA: percent agreement
RQ: research question
WHO: World Health Organization
