# Supplementary material for: Temporal and Location Variations, and Link Categories for the Dissemination of COVID-19–Related Information on Twitter During the SARS-CoV-2 Outbreak in Europe: Infoveillance Study
Source: J Med Internet Res. 2020 Aug 28;22(8):e19629. doi: 10.2196/19629 (PMC7470238; doi:10.2196/19629)
Supplement: Multimedia Appendix 1 [file jmir_v22i8e19629_app1.docx]

## Multimedia Appendix 1: Listing of all hashtags included in the study

Based on the global Twitter trends and media coverage in January 2020, eight hashtags were initially included for collecting tweets beginning on February, 9th 2020:

1. #nCov2019,
2. #nCov19,
3. #nCov,
4. #2019nCov,
5. #coronavirus,
6. #CoronaOutbreak,
7. #WuhanVirus,
8. #Wuhan.

Shortly after the WHO announcement on the naming of the virus and the related disease [1], the following two hashtags were included:

1. #covid19,
2. #sarscov2.

Further monitoring of Twitter disease-specific activities and reports in news outlets revealed that six hashtags:

1. #COVIDー19,
2. #coronavirusitaly,
3. #coronavirusitalia,
4. #CoronaVirusUpdates,
5. #CoronaVirusUpdate,
6. #Covid_19

were increasingly used and therefore included in the study setting in late February and at the beginning of March 2020.
