# Supplementary material for: Temporal and Location Variations, and Link Categories for the Dissemination of COVID-19–Related Information on Twitter During the SARS-CoV-2 Outbreak in Europe: Infoveillance Study
Source: J Med Internet Res. 2020 Aug 28;22(8):e19629. doi: 10.2196/19629 (PMC7470238; doi:10.2196/19629)
Supplement: Multimedia Appendix 2 [file jmir_v22i8e19629_app2.docx]

## Multimedia Appendix 2: Temporal variations of hashtag frequencies between 2020-02-09 and 2020-04-11


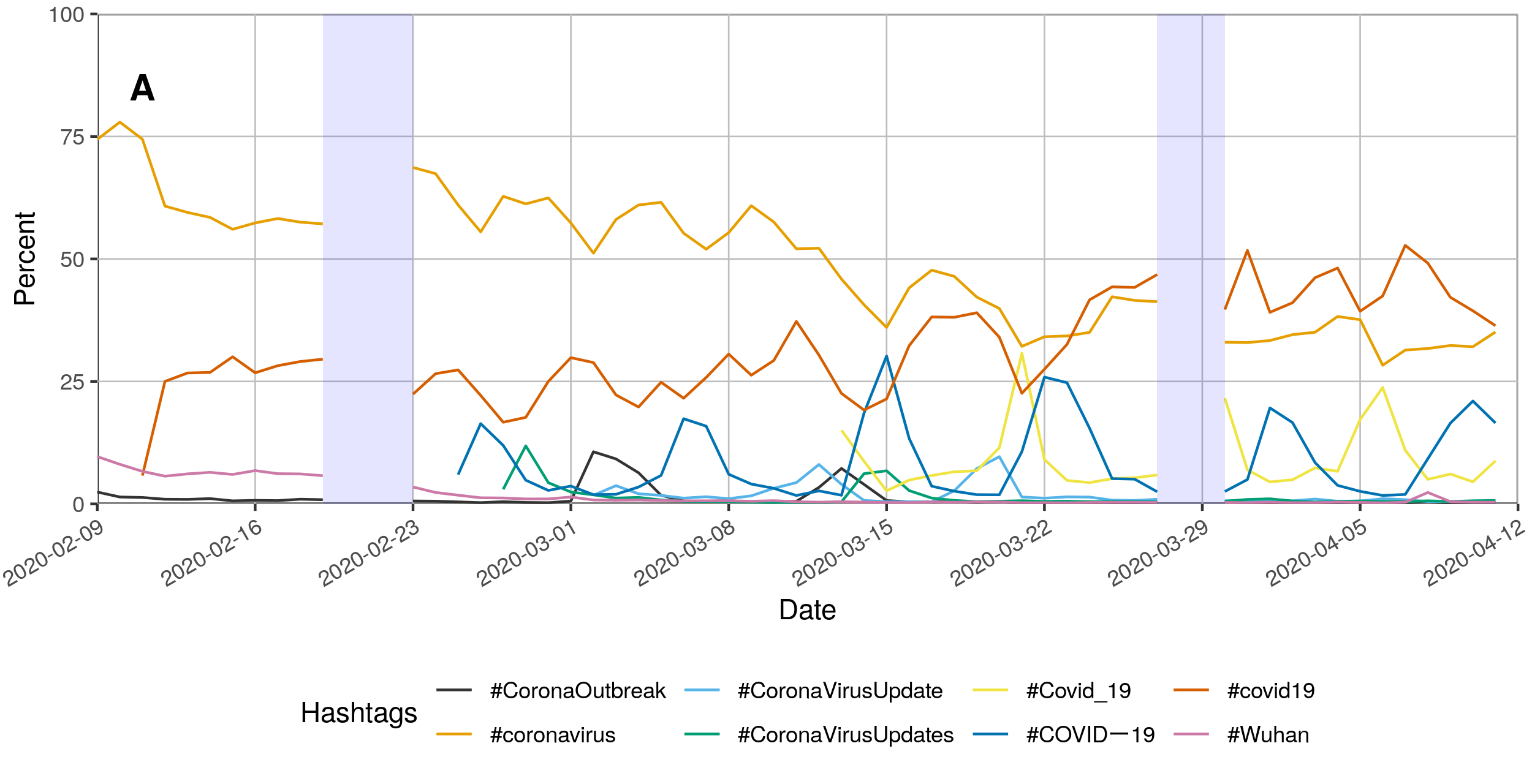


Figure 1: The relative proportion of tweets with the 1st to 8th rank hashtag. The capital letter ‘A’ represents the naming of the disease by the WHO on 11th February 2020. Blue rectangle: No tweets were collected between 20th February and 22nd February as well as between 28th March and 29th March due to technical issues.


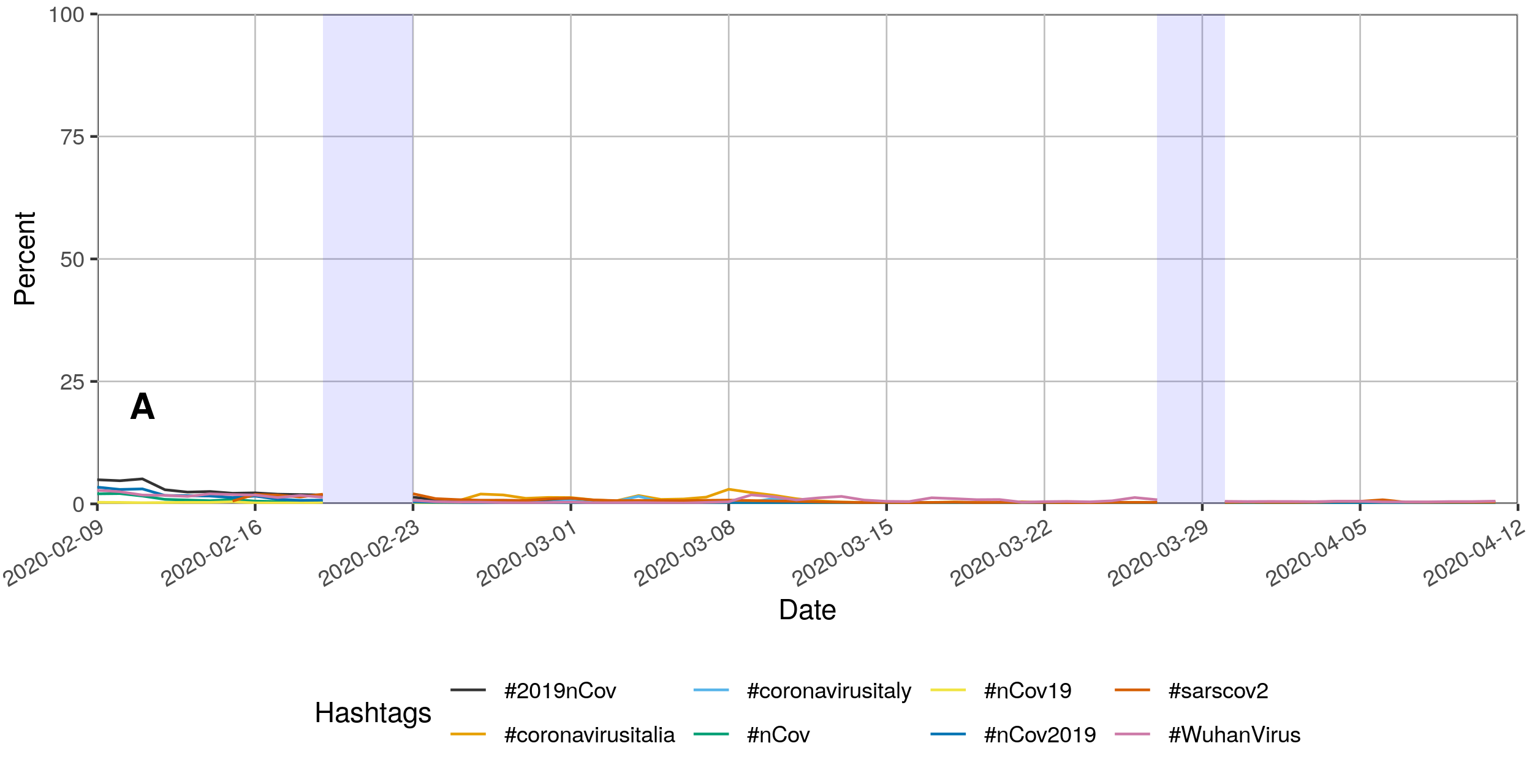


Figure 2: The relative proportion of tweets with the 9th to 16th rank hashtag. The capital letter ‘A’ represents the naming of the disease by the WHO on 11th February 2020. Blue rectangle: No tweets were collected between 20th February and 22nd February as well as between 28th March and 29th March due to technical issues.


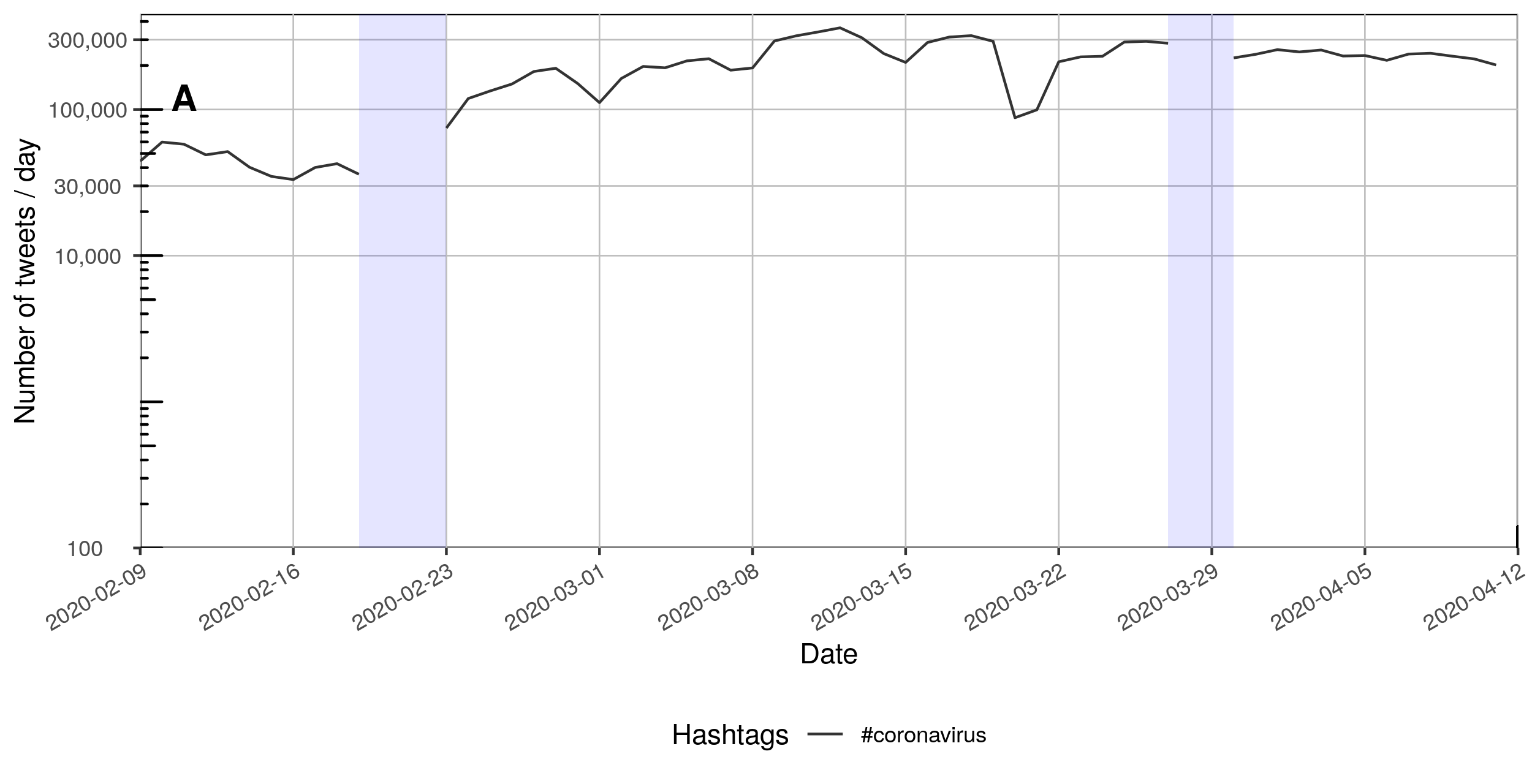


Figure 3: Number of tweets with hashtag ‘#coronavirus’ (1st rank) between 9th February 2020 and 11th April 2020. The capital letter ‘A’ represents the naming of the disease by the WHO on 11th February 2020. Blue rectangle: No tweets were collected between 20th February and 22nd February as well as between 28th March and 29th March due to technical issues.


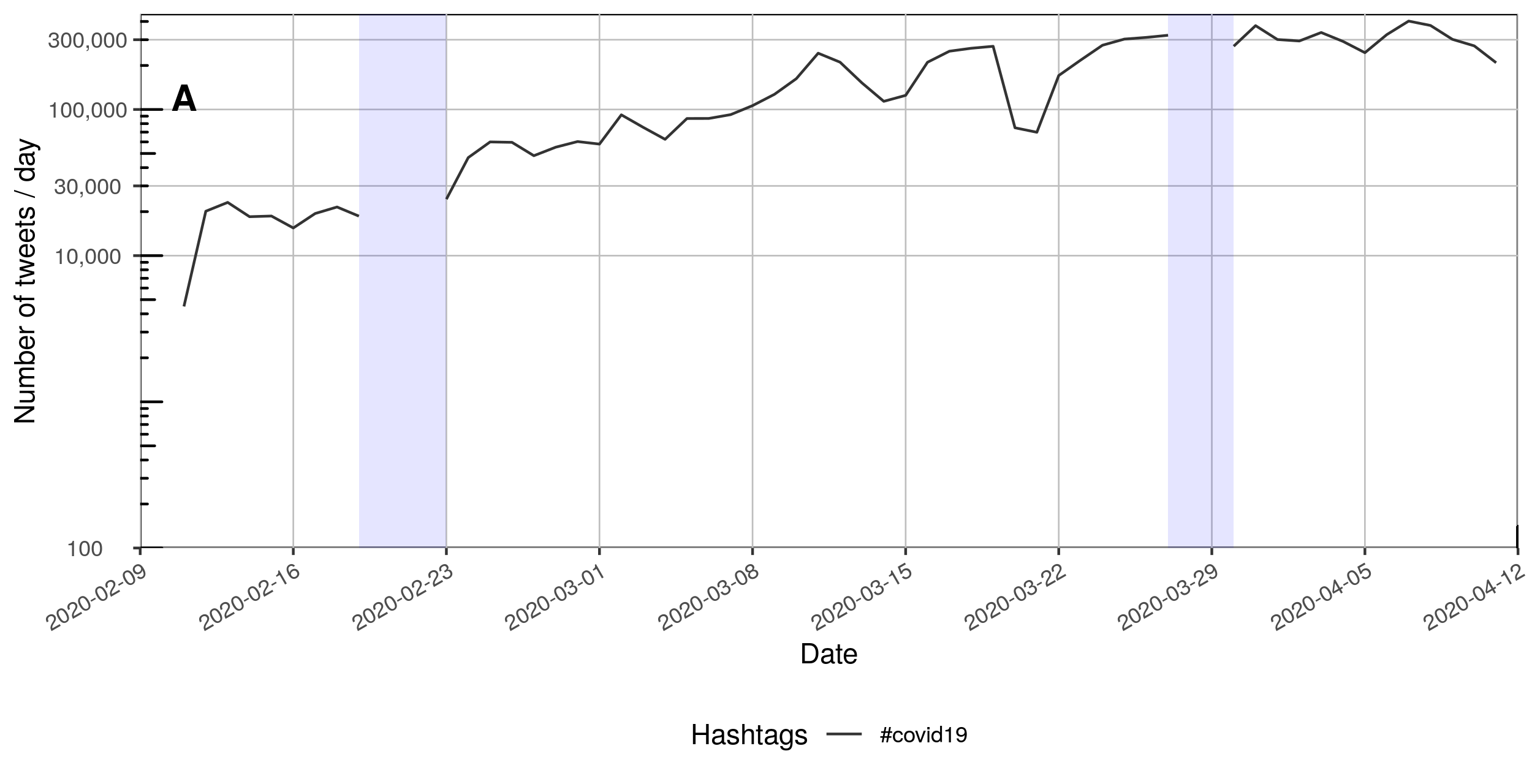


Figure 4: Number of tweets with hashtag ‘#covid19 (2nd rank) between 9th February 2020 and 11th April 2020. The capital letter ‘A’ represents the naming of the disease by the WHO on 11th February 2020. Blue rectangle: No tweets were collected between 20th February and 22nd February as well as between 28th March and 29th March due to technical issues.


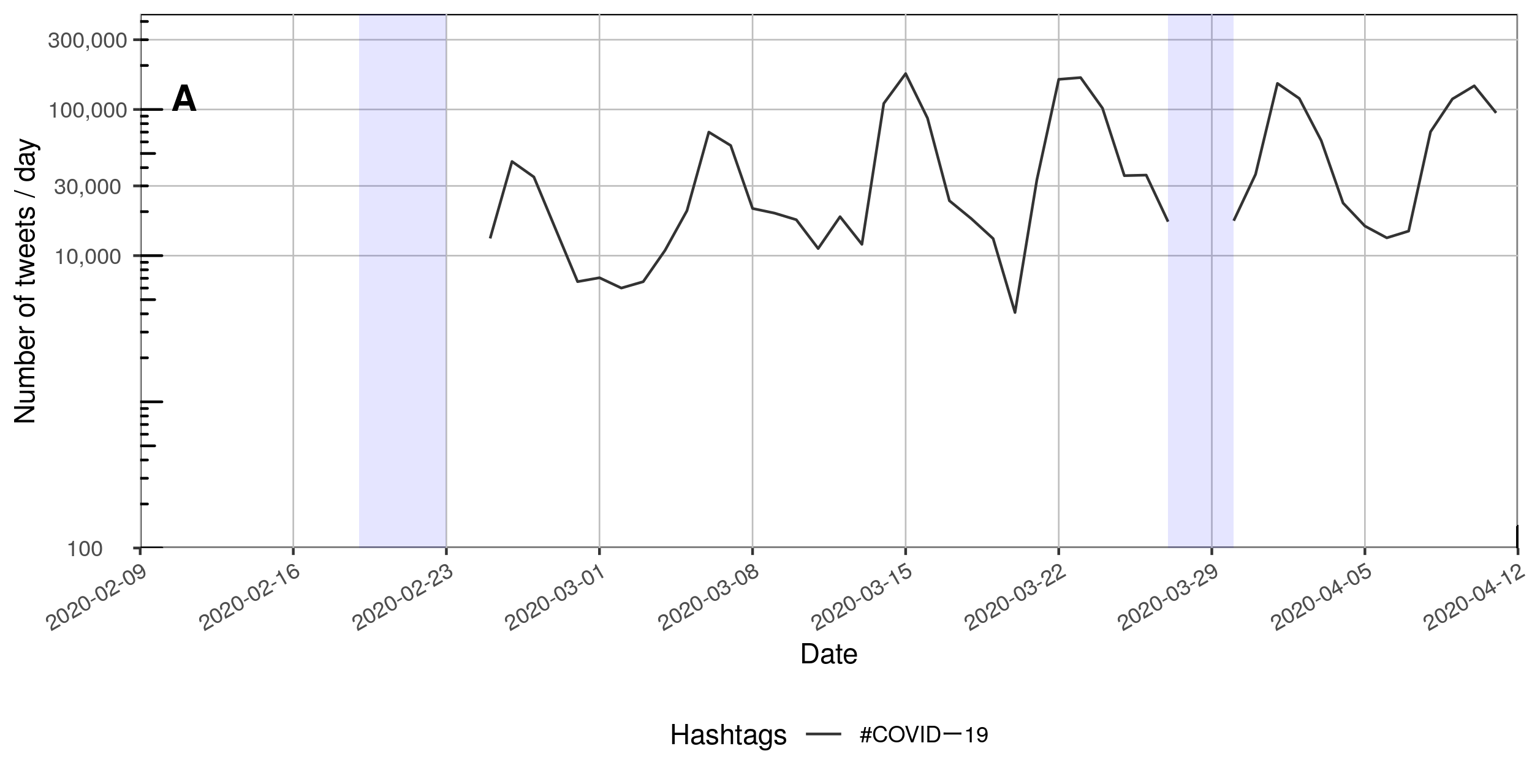


Figure 5: Number of tweets with hashtag ‘#COVID—19’ (3rd rank) between 9th February 2020 and 11th April 2020. The capital letter ‘A’ represents the naming of the disease by the WHO on 11th February 2020. Blue rectangle: No tweets were collected between 20th February and 22nd February as well as between 28th March and 29th March due to technical issues.


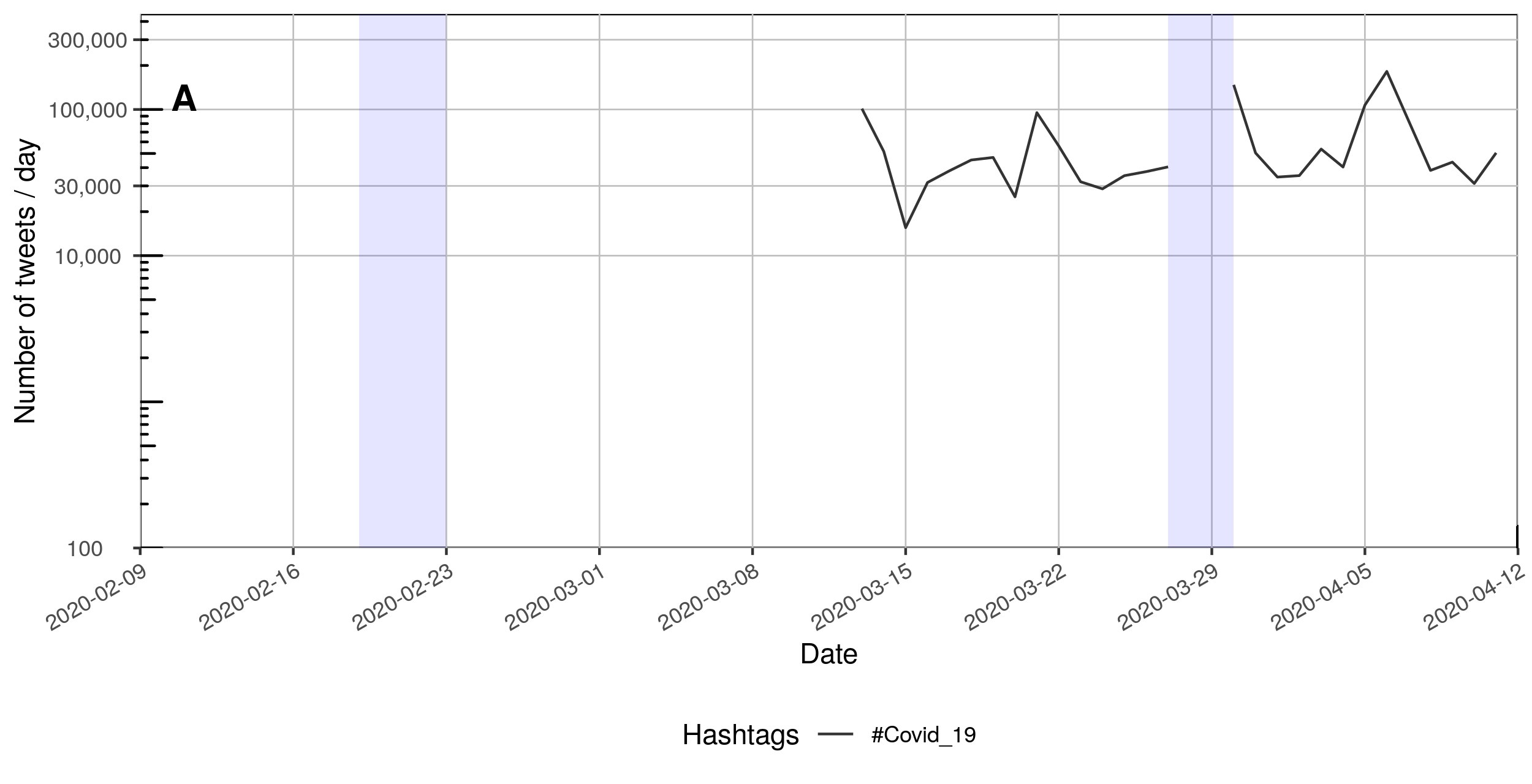


Figure 6: Number of tweets with hashtag ‘#Covid_19’ (4th rank) between 9th February 2020 and 11th April 2020. The capital letter ‘A’ represents the naming of the disease by the WHO on 11th February 2020. Blue rectangle: No tweets were collected between 20th February and 22nd February as well as between 28th March and 29th March due to technical issues.


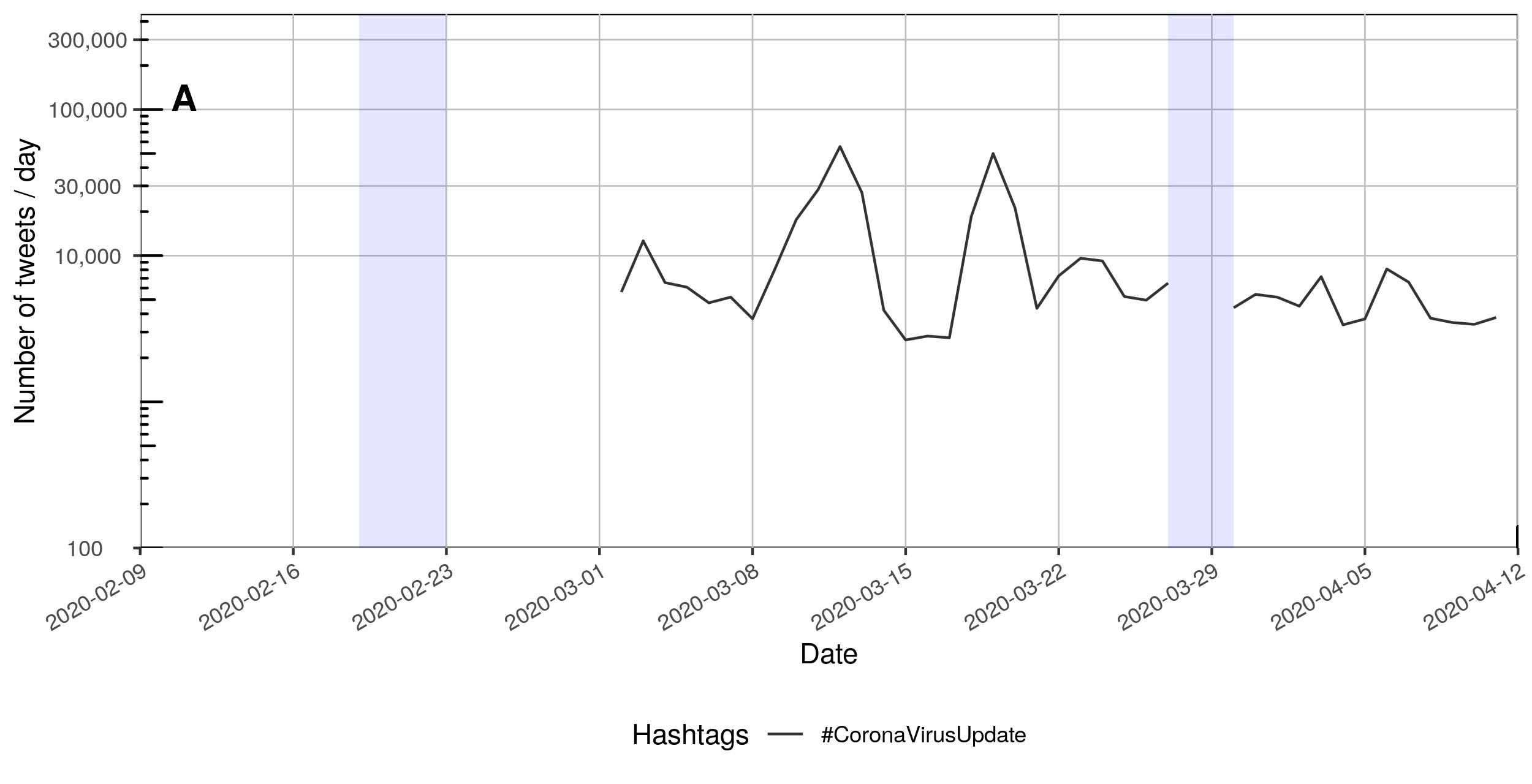


Figure 7: Number of tweets with hashtag ‘#CoronaVirusUpdate’ (5th rank) between 9th February 2020 and 11th April 2020. The capital letter ‘A’ represents the naming of the disease by the WHO on 11th February 2020. Blue rectangle: No tweets were collected between 20th February and 22nd February as well as between 28th March and 29th March due to technical issues.


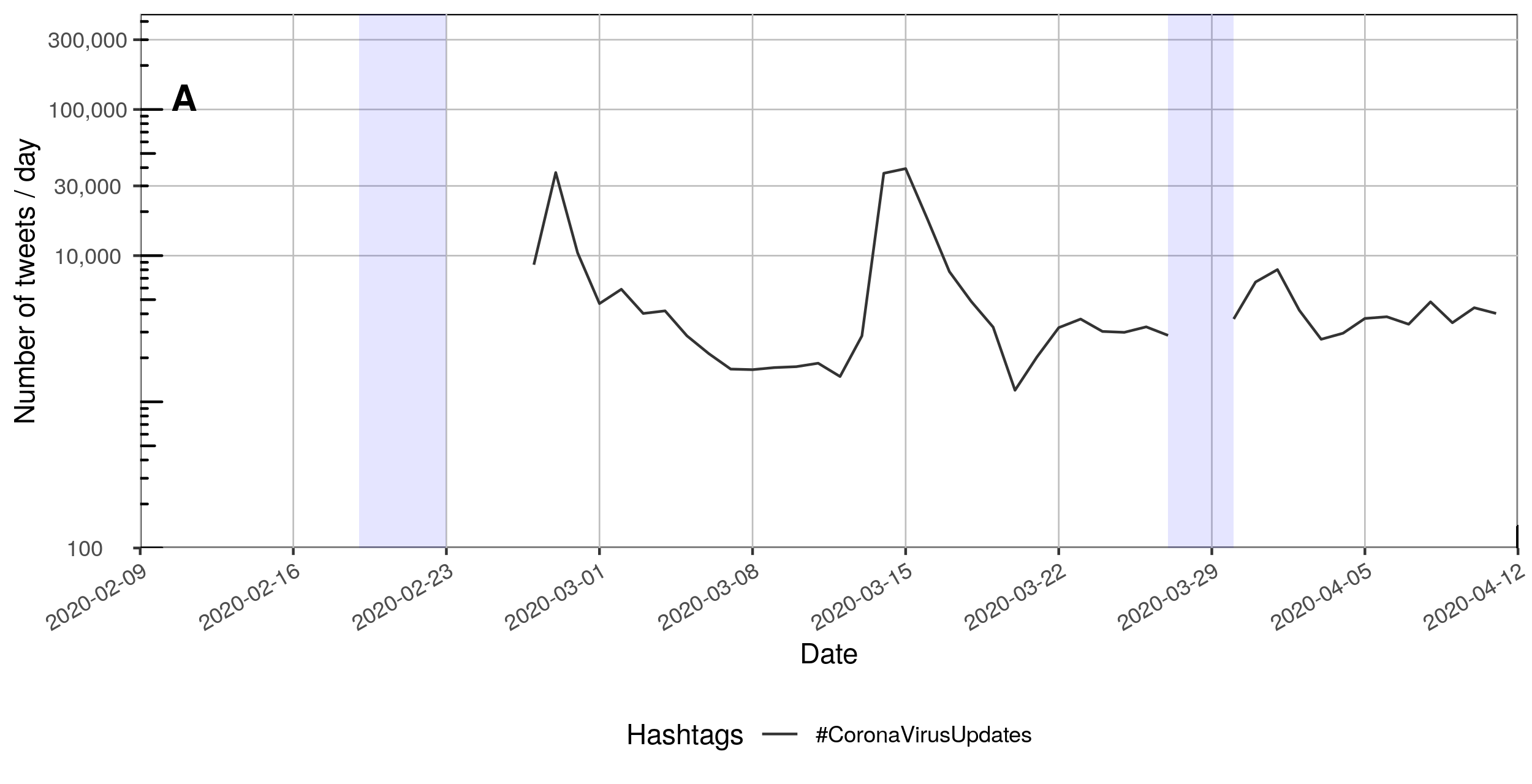


Figure 8: Number of tweets with hashtag ‘#CoronaVirusUpdates’ (6th rank) between 9th February 2020 and 11th April 2020. The capital letter ‘A’ represents the naming of the disease by the WHO on 11th February 2020. Blue rectangle: No tweets were collected between 20th February and 22nd February as well as between 28th March and 29th March due to technical issues.


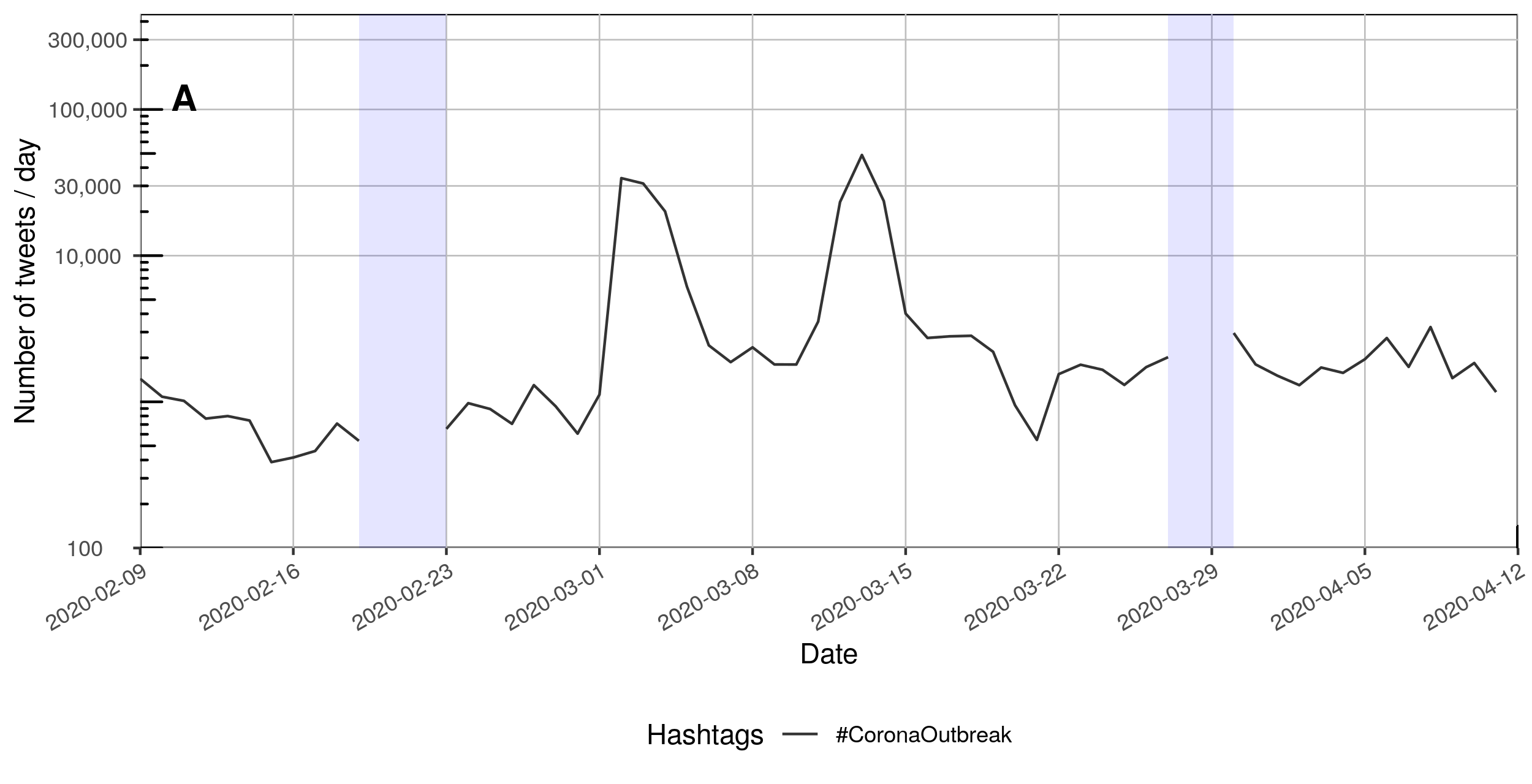


Figure 9: Number of tweets with hashtag ‘#CoronaOutbreak’ (7th rank) between 9th February 2020 and 11th April 2020. The capital letter ‘A’ represents the naming of the disease by the WHO on 11th February 2020. Blue rectangle: No tweets were collected between 20th February and 22nd February as well as between 28th March and 29th March due to technical issues.


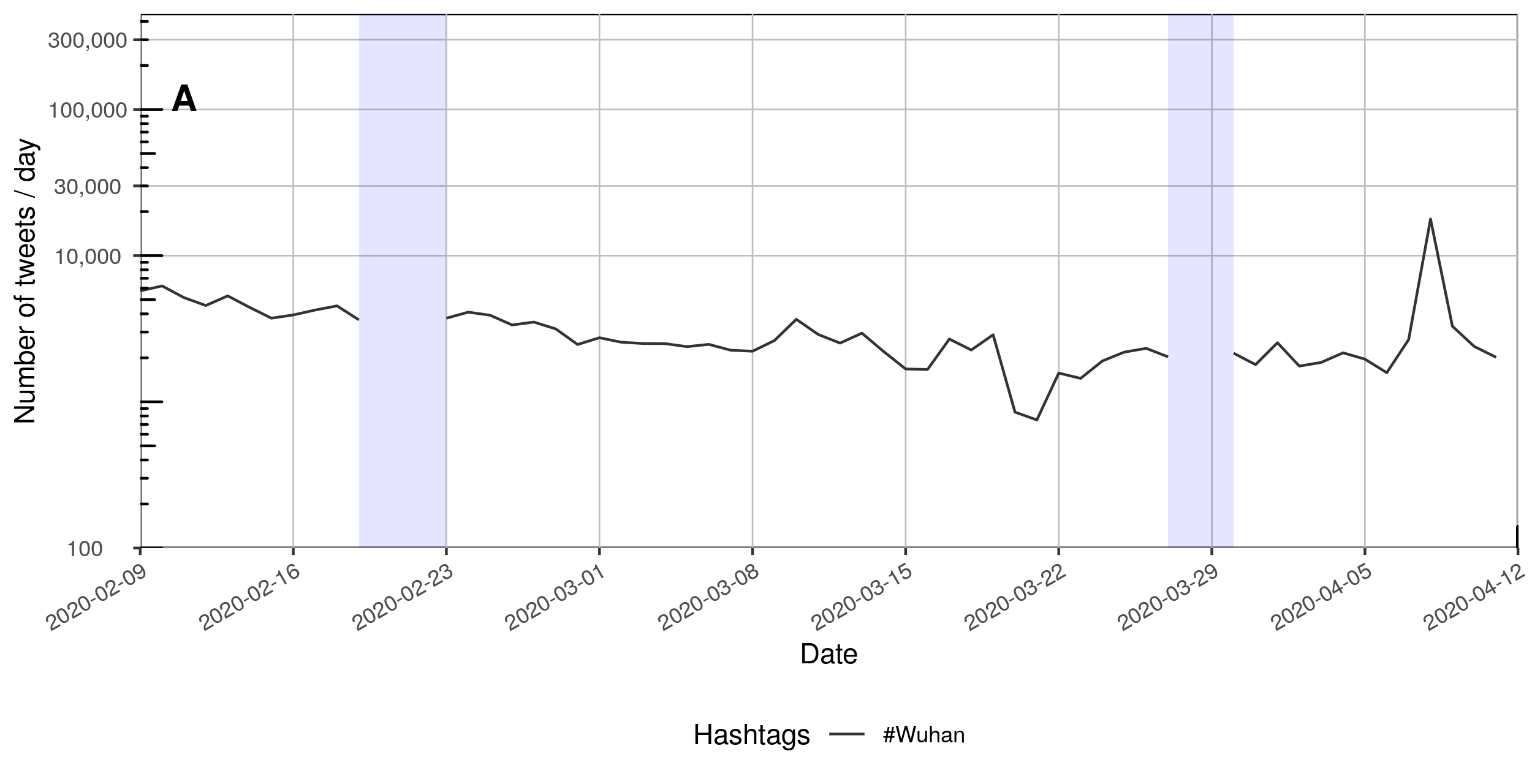


Figure 10: Number of tweets with hashtag ‘#Wuhan’ (8th rank) between 9th February 2020 and 11th April 2020. The capital letter ‘A’ represents the naming of the disease by the WHO on 11th February 2020. Blue rectangle: No tweets were collected between 20th February and 22nd February as well as between 28th March and 29th March due to technical issues.


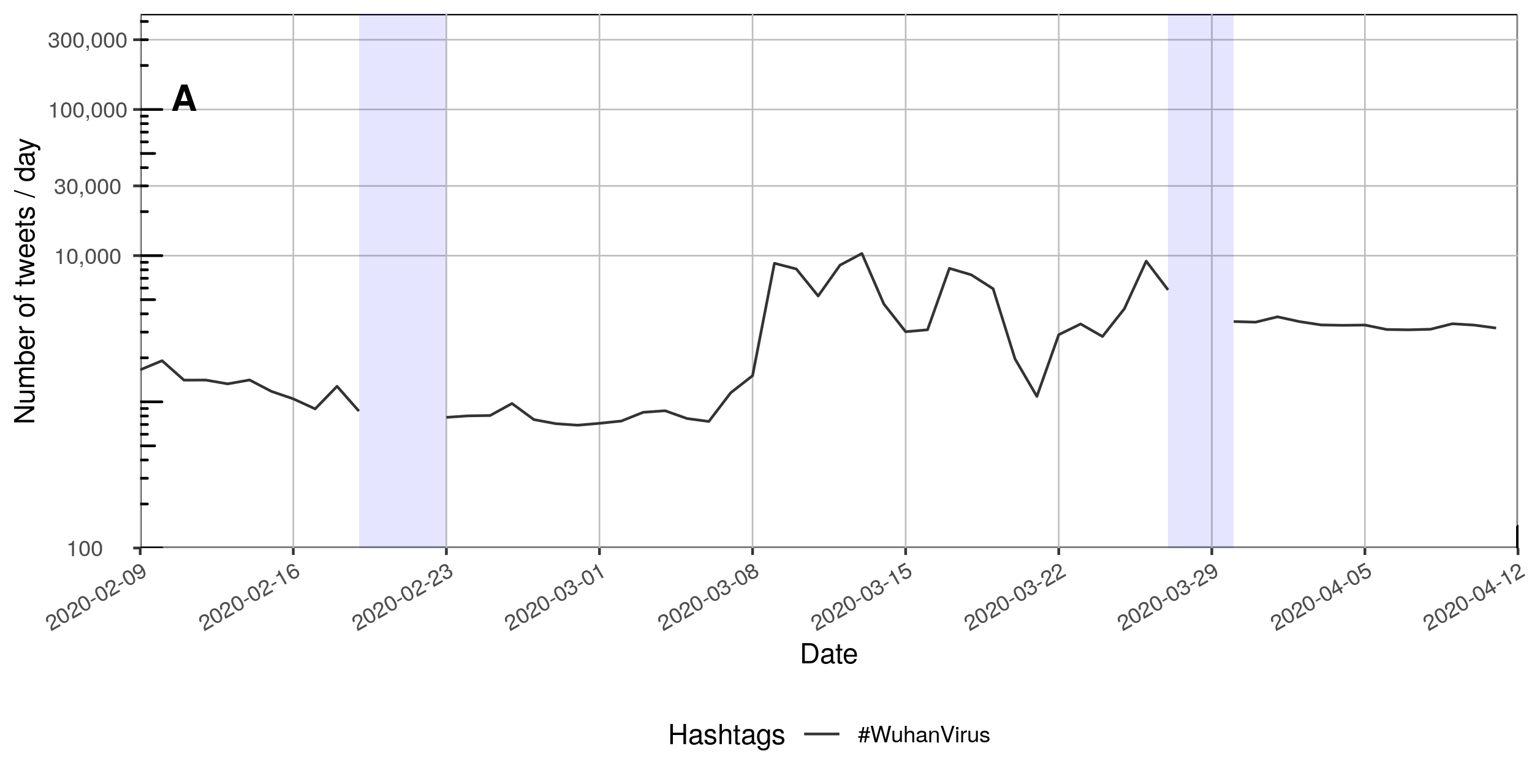


Figure 11: Number of tweets with hashtag ‘#WuhanVirus’ (9th rank) between 9th February 2020 and 11th April 2020. The capital letter ‘A’ represents the naming of the disease by the WHO on 11th February 2020. Blue rectangle: No tweets were collected between 20th February and 22nd February as well as between 28th March and 29th March due to technical issues.


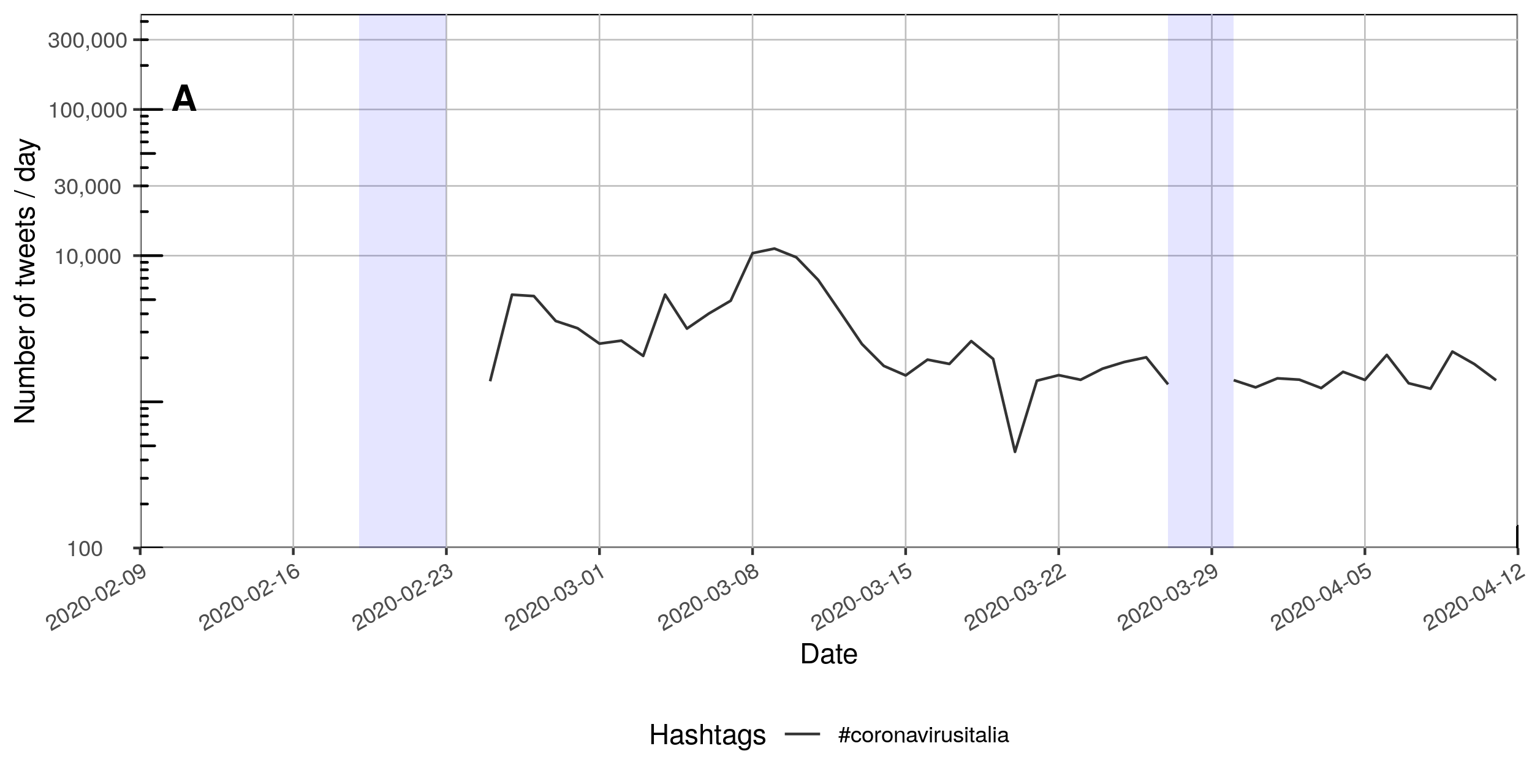


Figure 12: Number of tweets with hashtag ‘#coronavirusitalia’ (10th rank) between 9th February 2020 and 11th April 2020. The capital letter ‘A’ represents the naming of the disease by the WHO on 11th February 2020. Blue rectangle: No tweets were collected between 20th February and 22nd February as well as between 28th March and 29th March due to technical issues.


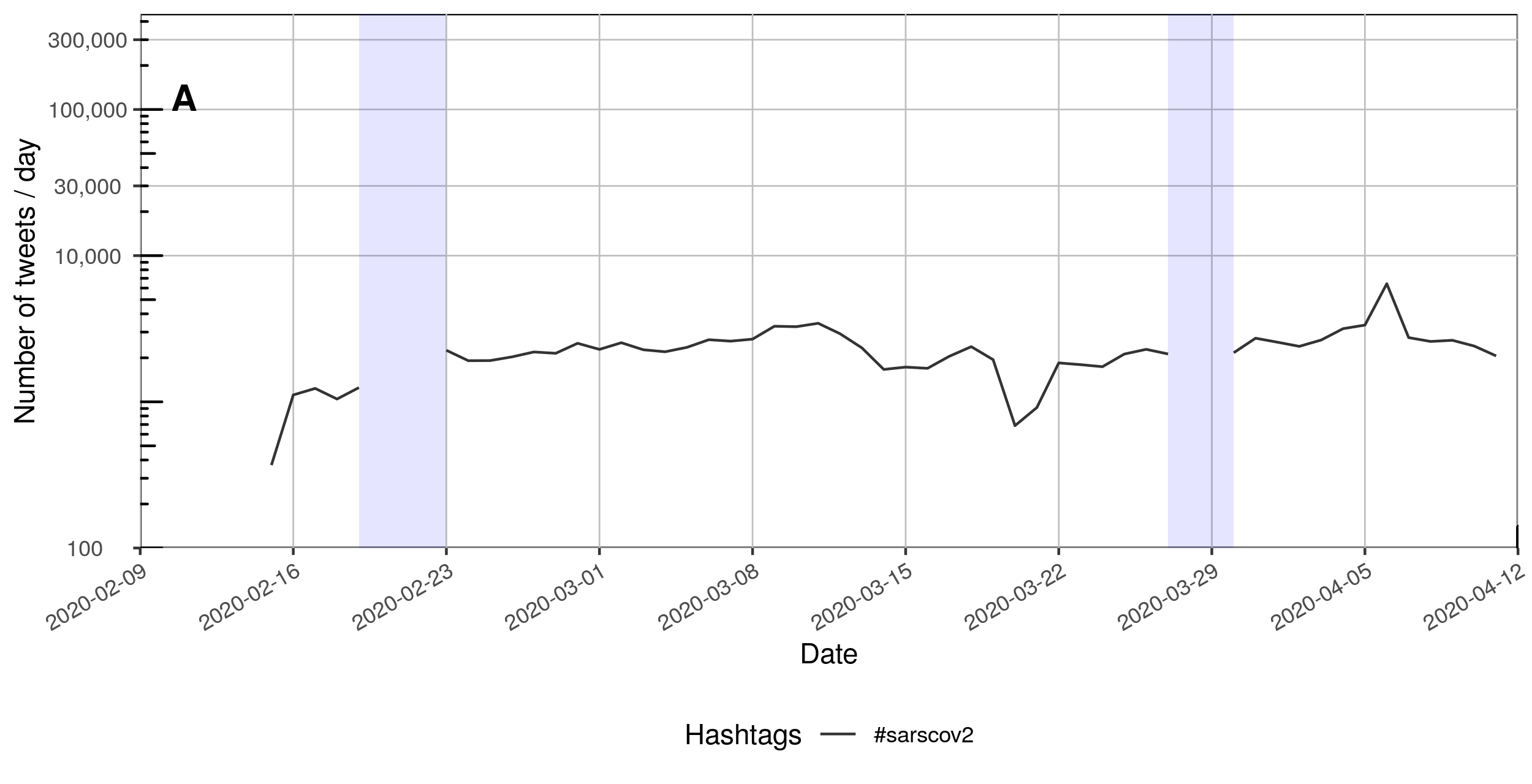


Figure 13: Number of tweets with hashtag ‘#sarscov2’ (11th rank) between 9th February 2020 and 11th April 2020. The capital letter ‘A’ represents the naming of the disease by the WHO on 11th February 2020. Blue rectangle: No tweets were collected between 20th February and 22nd February as well as between 28th March and 29th March due to technical issues.


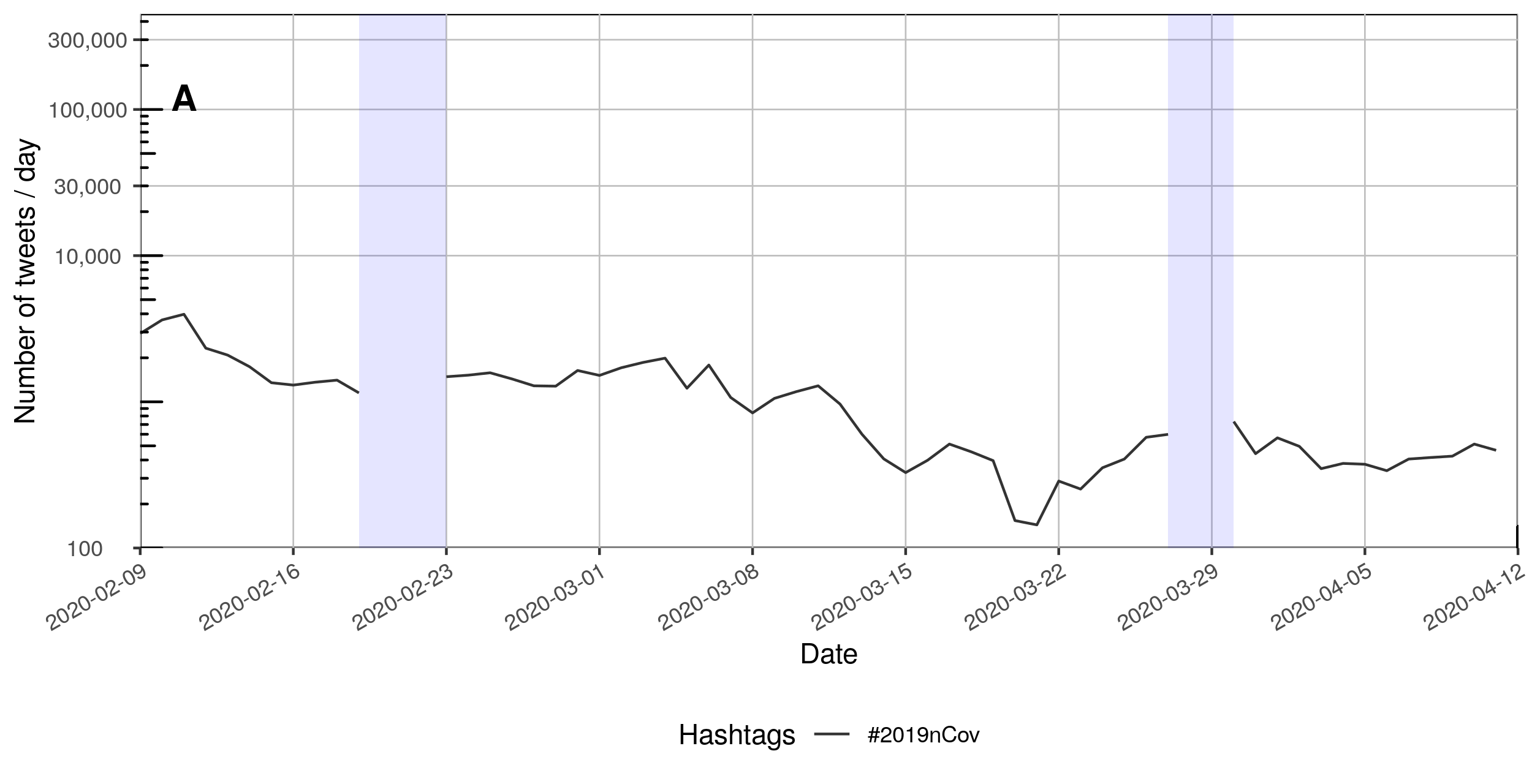


Figure 14: Number of tweets with hashtag ‘#2019nCov’ (12th rank) between 9th February 2020 and 11th April 2020. The capital letter ‘A’ represents the naming of the disease by the WHO on 11th February 2020. Blue rectangle: No tweets were collected between 20th February and 22nd February as well as between 28th March and 29th March due to technical issues.


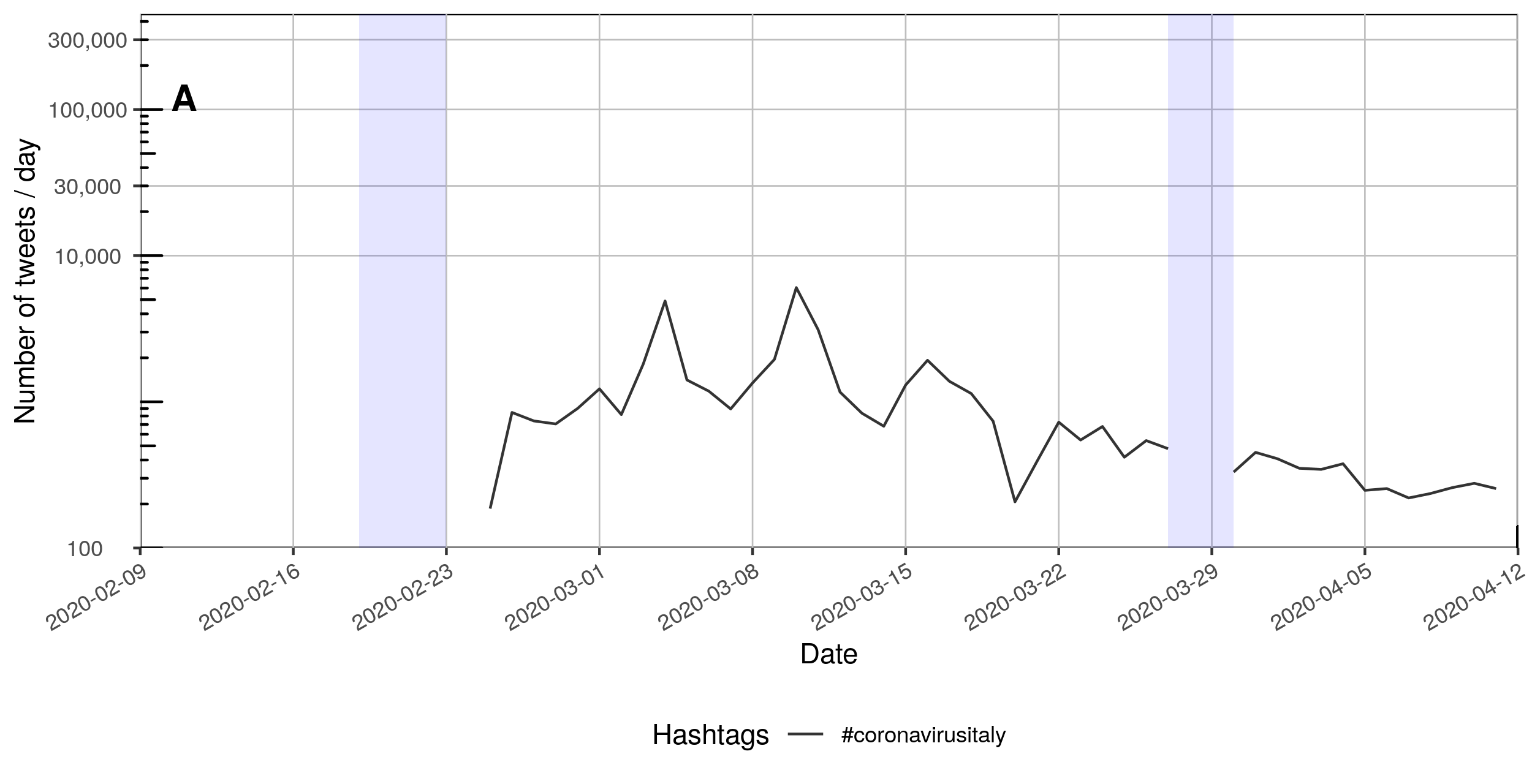


Figure 15: Number of tweets with hashtag ‘#coronavirusitaly’ (13th rank) between 9th February 2020 and 11th April 2020. The capital letter ‘A’ represents the naming of the disease by the WHO on 11th February 2020. Blue rectangle: No tweets were collected between 20th February and 22nd February as well as between 28th March and 29th March due to technical issues.


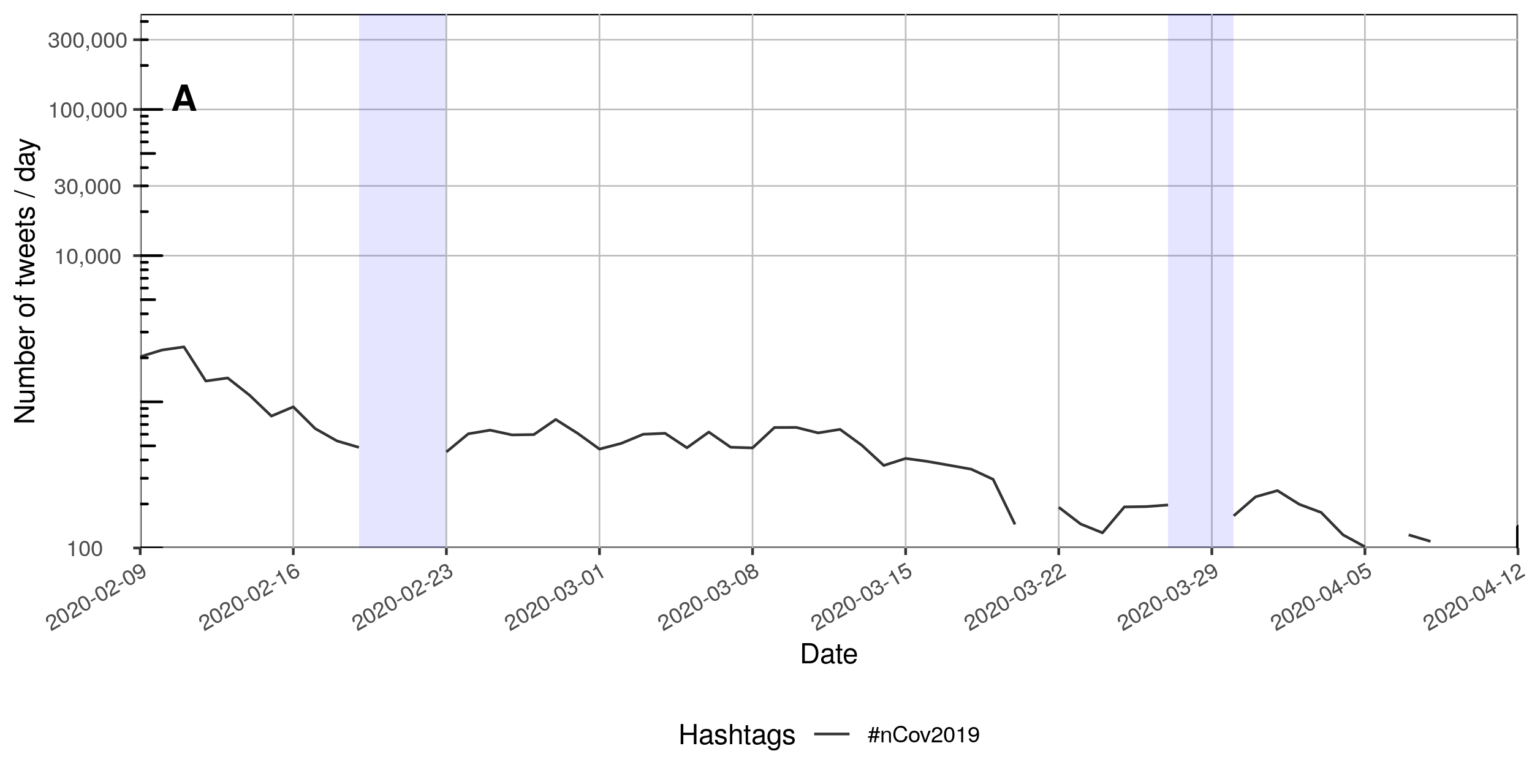


Figure 16: Number of tweets with hashtag ‘#nCov2019’ (14th rank) between 9th February 2020 and 11th April 2020. The capital letter ‘A’ represents the naming of the disease by the WHO on 11th February 2020. Blue rectangle: No tweets were collected between 20th February and 22nd February as well as between 28th March and 29th March due to technical issues.


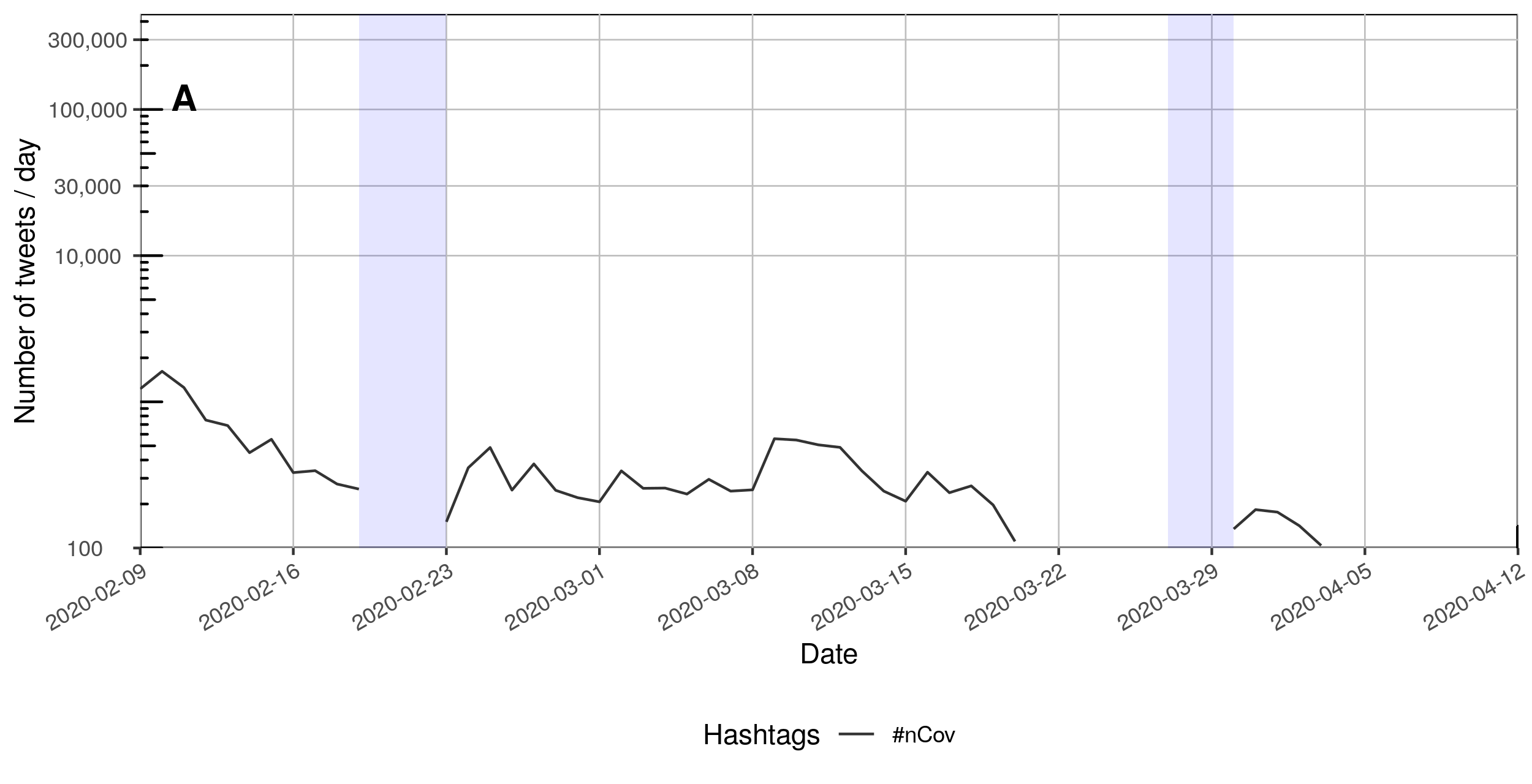


Figure 17: Number of tweets with hashtag ‘#nCov’ (15th rank) between 9th February 2020 and 11th April 2020. The capital letter ‘A’ represents the naming of the disease by the WHO on 11th February 2020. Blue rectangle: No tweets were collected between 20th February and 22nd February as well as between 28th March and 29th March due to technical issues.


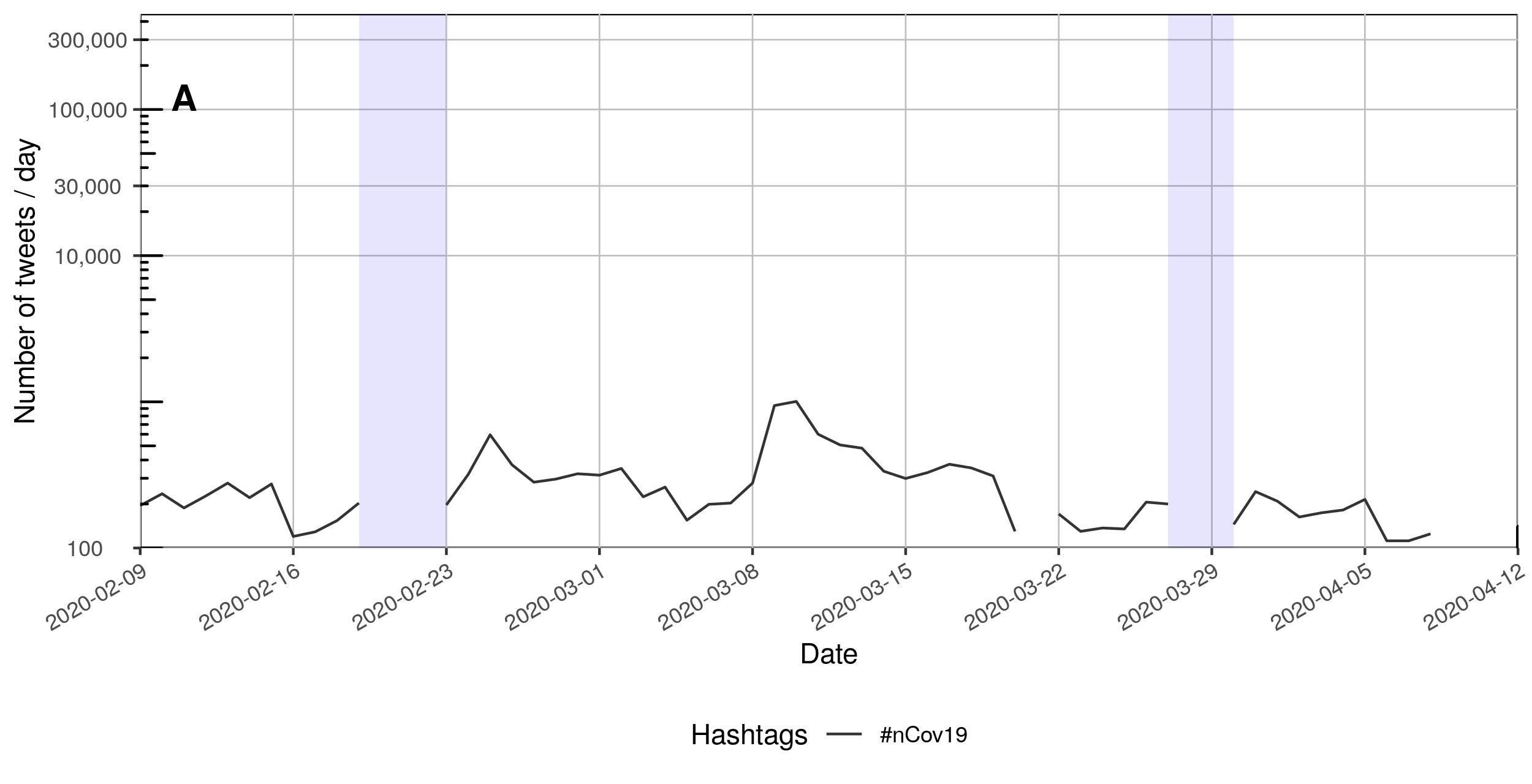


Figure 18: Number of tweets with hashtag ‘#nCov19’ (16th rank) between 9th February 2020 and 11th April 2020. The capital letter ‘A’ represents the naming of the disease by the WHO on 11th February 2020. Blue rectangle: No tweets were collected between 20th February and 22nd February as well as between 28th March and 29th March due to technical issues.
