# Supplementary material for: Temporal and Location Variations, and Link Categories for the Dissemination of COVID-19–Related Information on Twitter During the SARS-CoV-2 Outbreak in Europe: Infoveillance Study
Source: J Med Internet Res. 2020 Aug 28;22(8):e19629. doi: 10.2196/19629 (PMC7470238; doi:10.2196/19629)

## Multimedia Appendix 4: Seven-day interval plots of the geographical variation of tweets in the European Countries
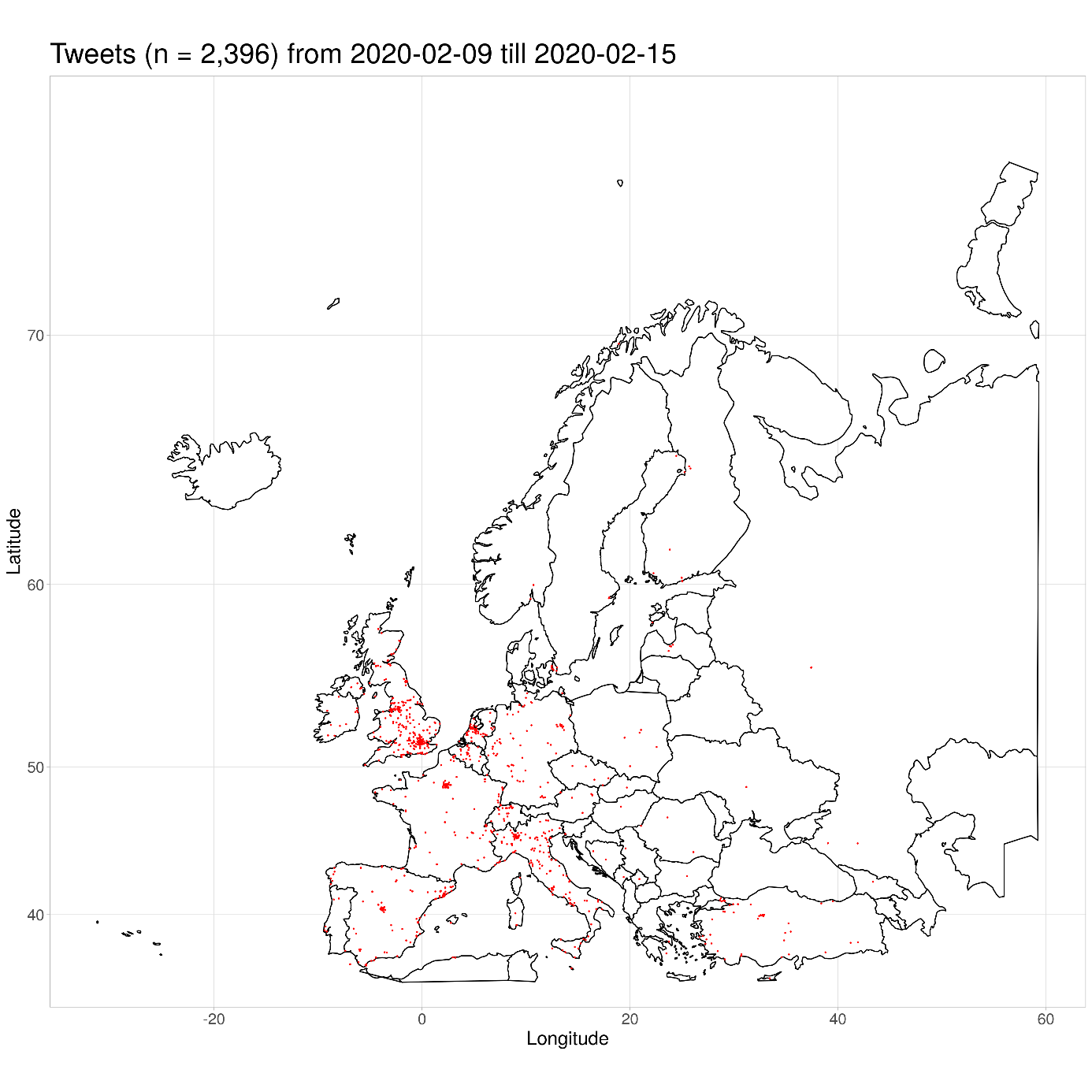

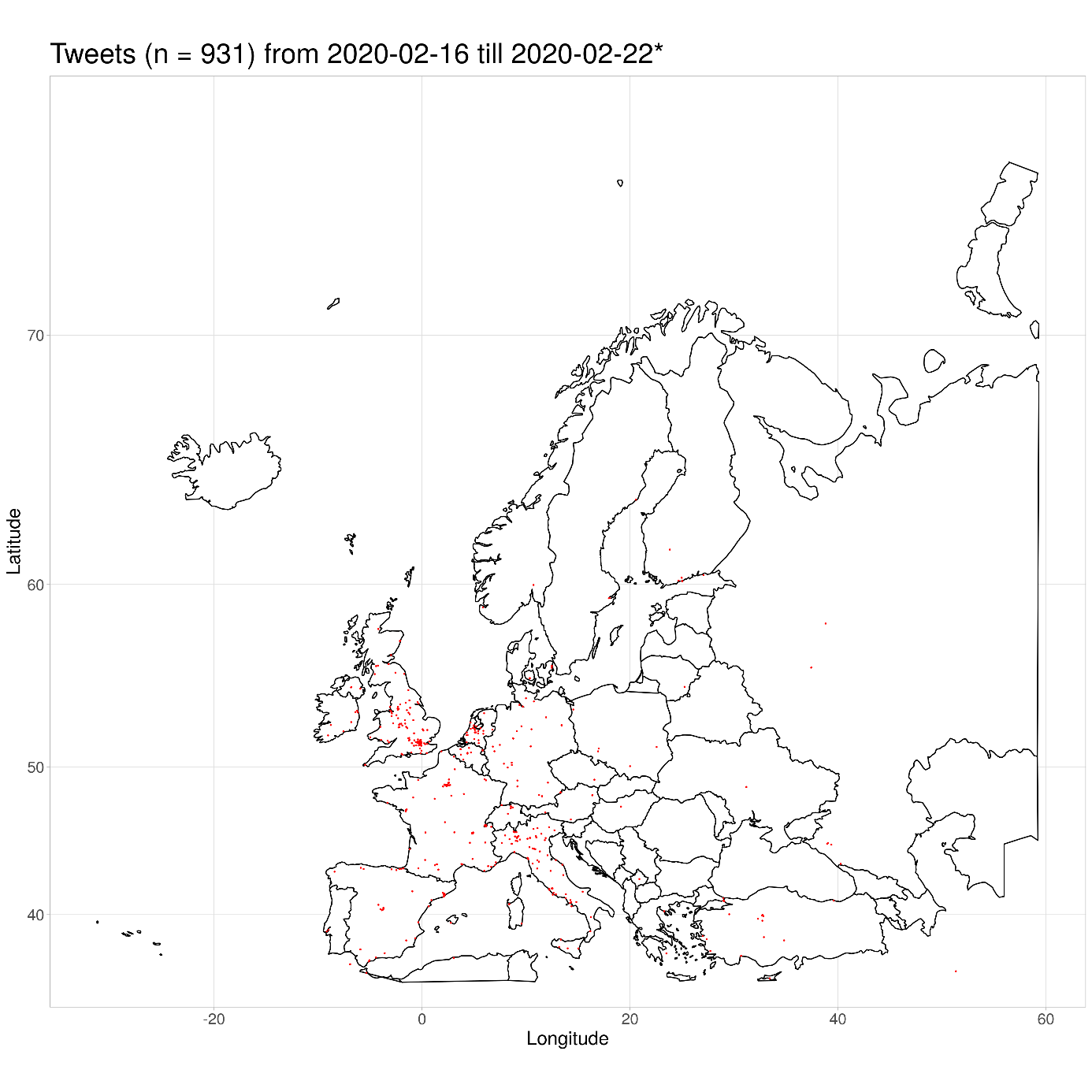

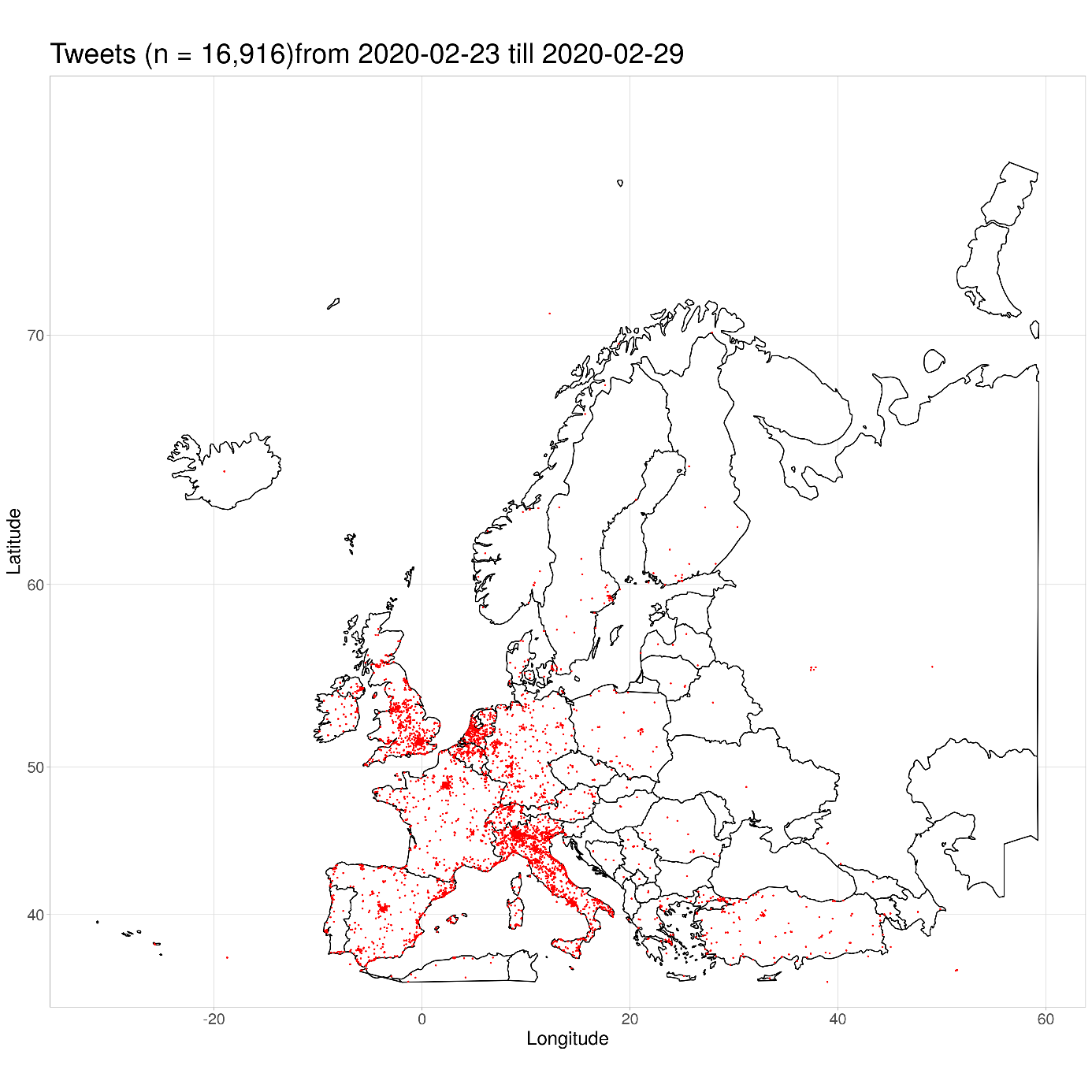

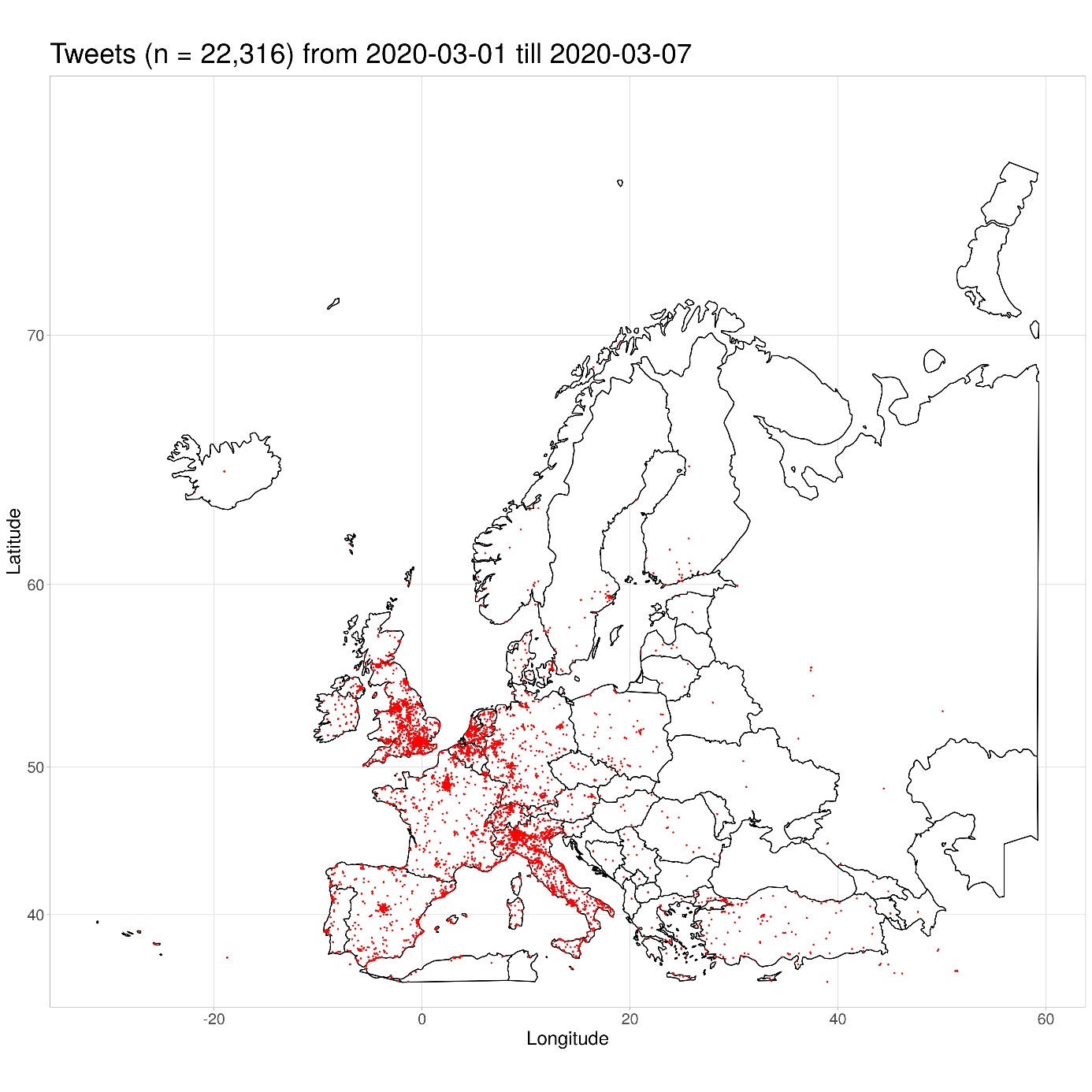

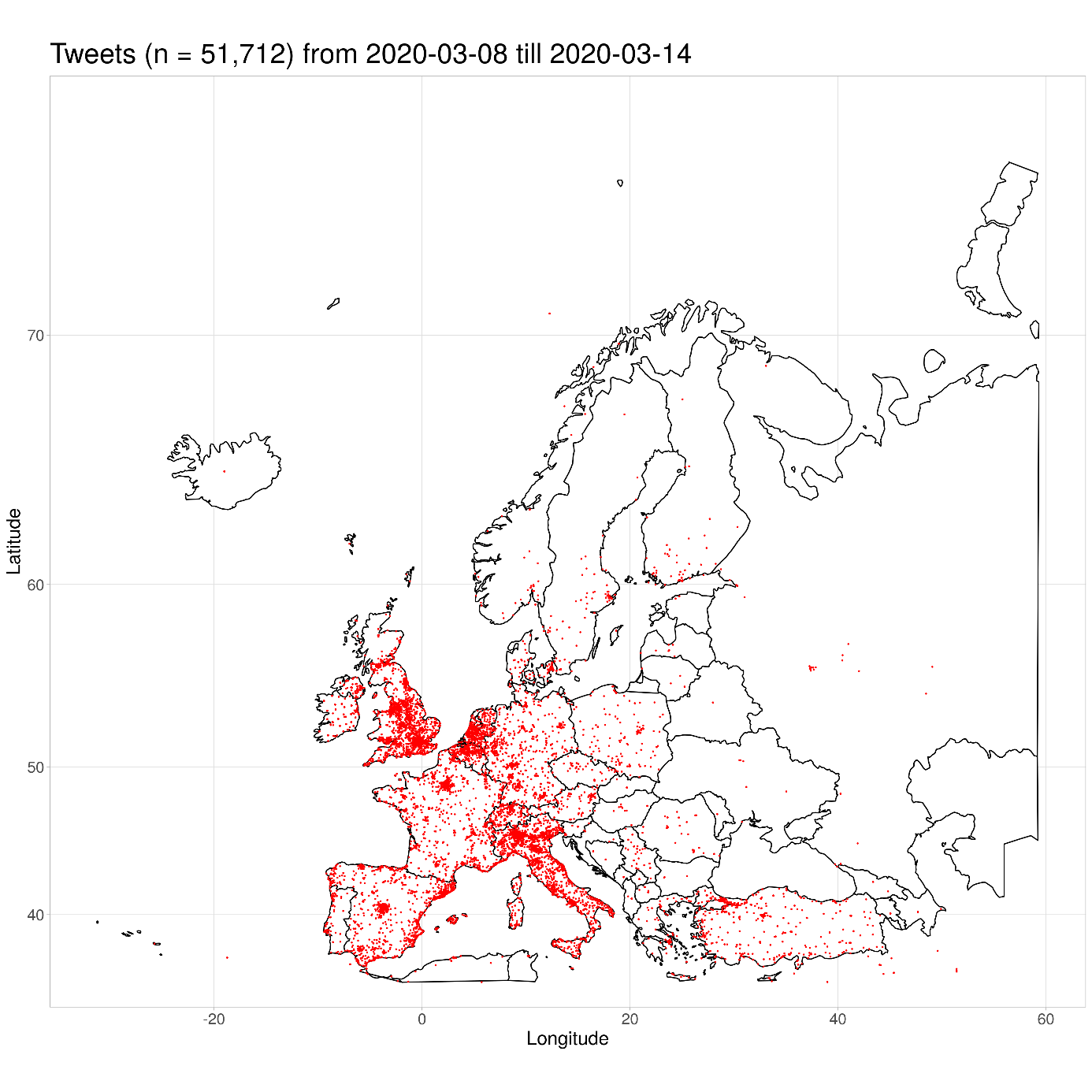

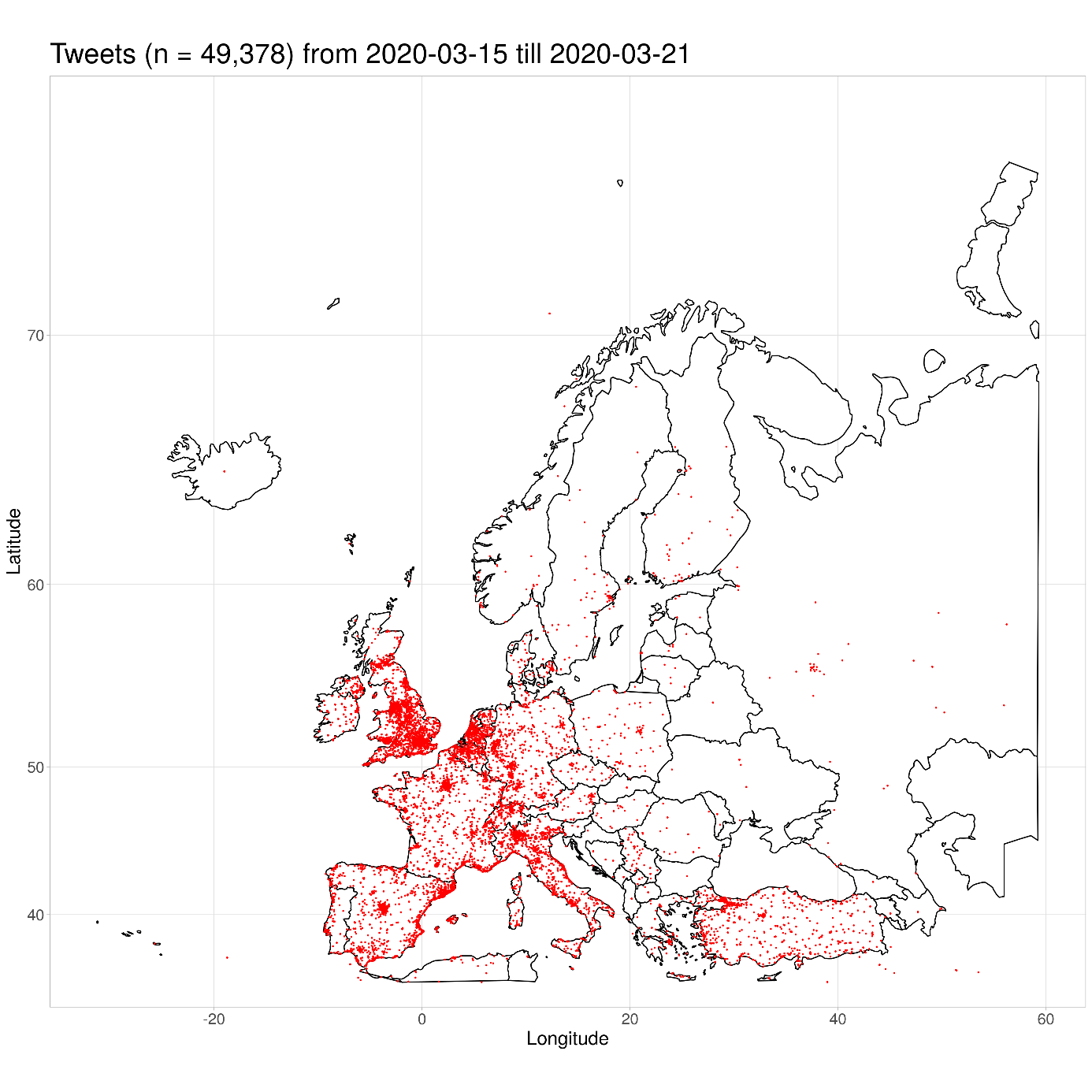

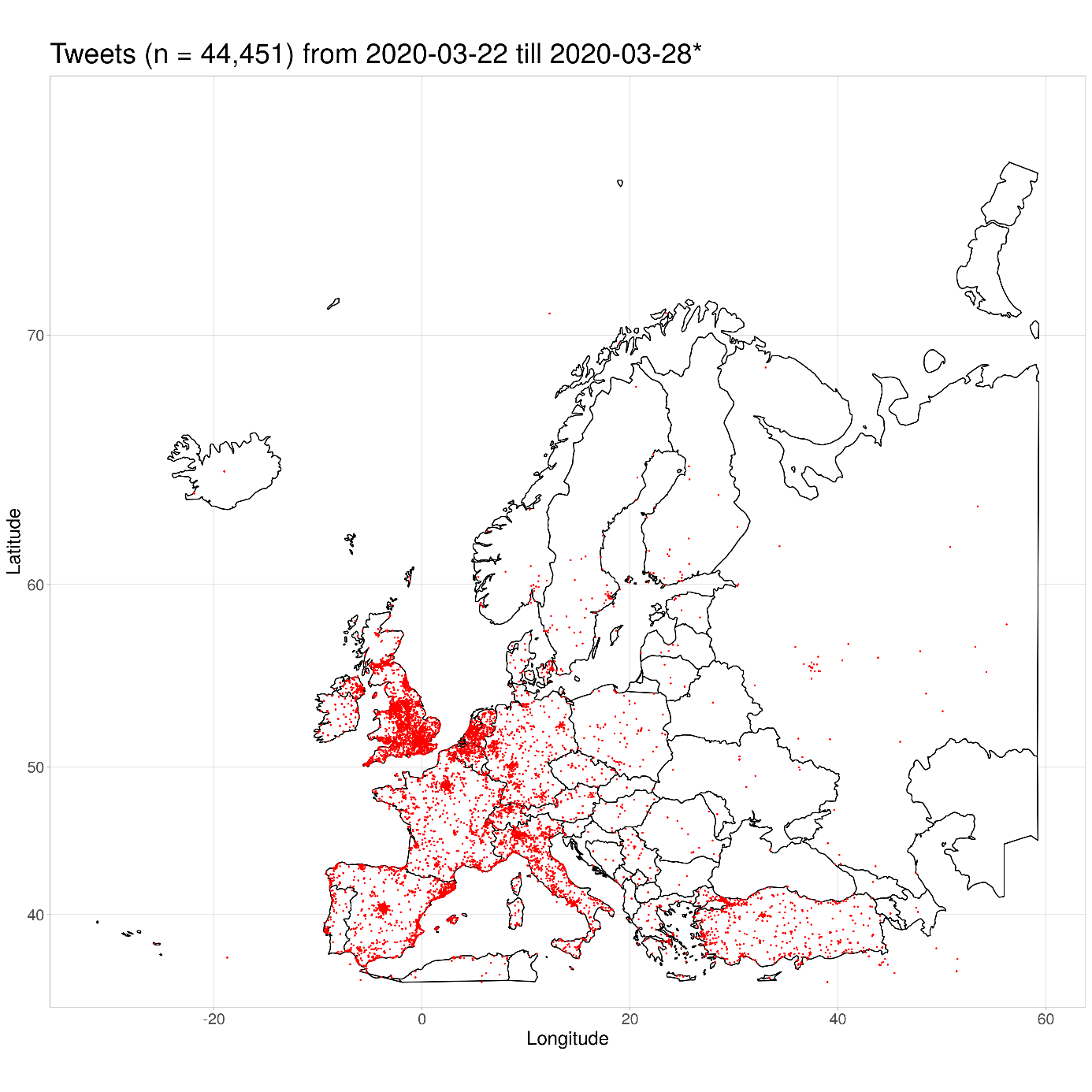

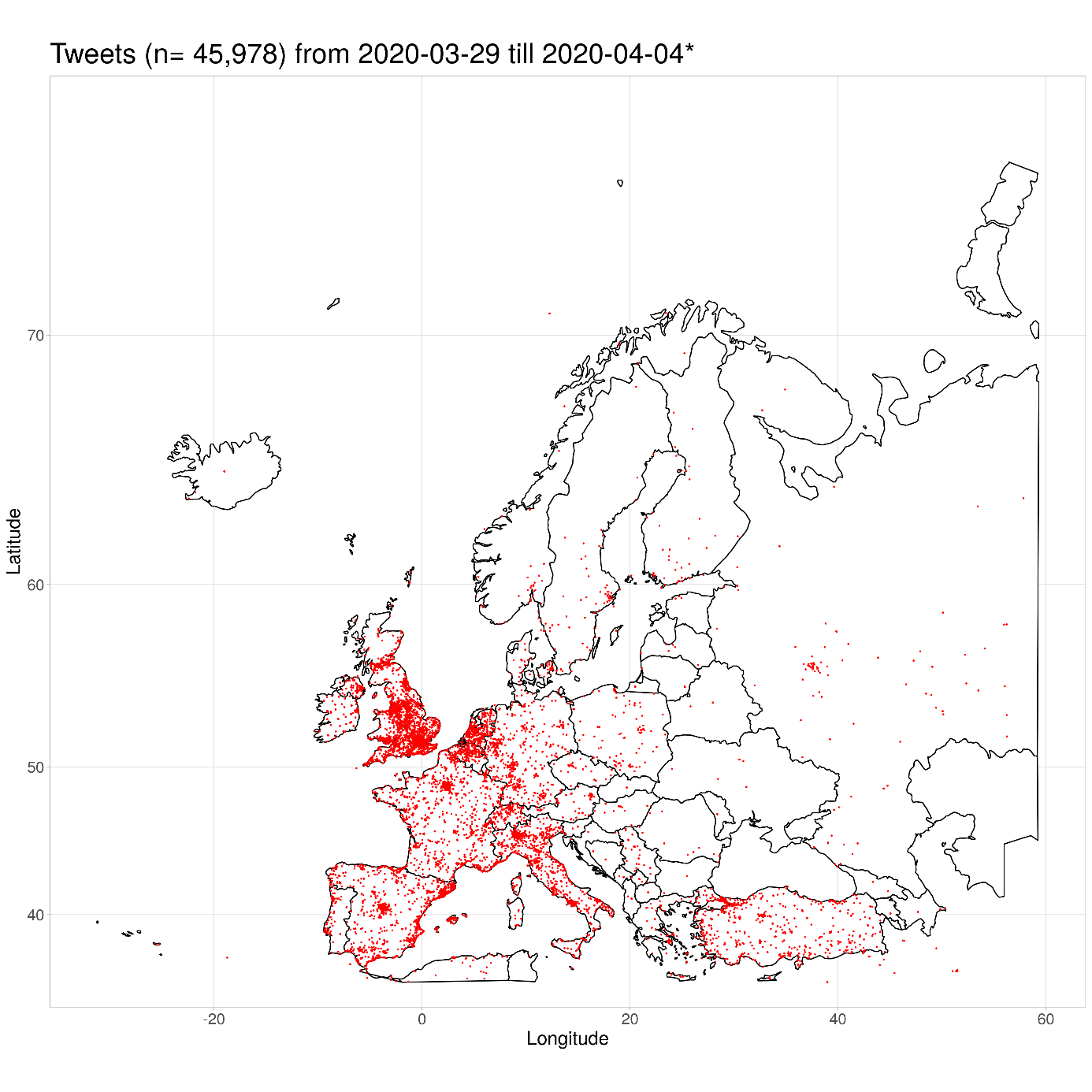

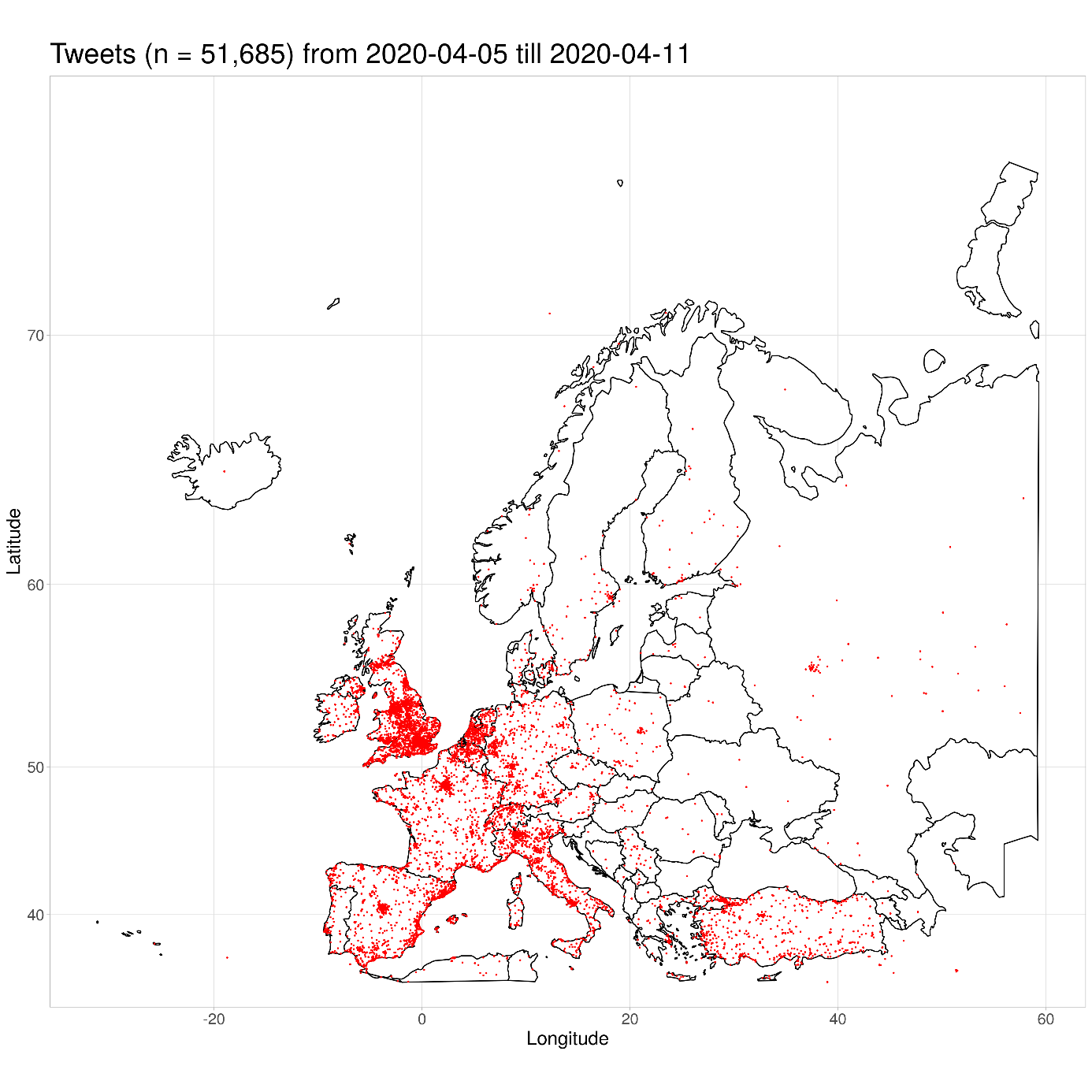

Supplement: Multimedia Appendix 4 [file jmir_v22i8e19629_app4.docx]
