# Supplementary material for: Temporal and Location Variations, and Link Categories for the Dissemination of COVID-19–Related Information on Twitter During the SARS-CoV-2 Outbreak in Europe: Infoveillance Study
Source: J Med Internet Res. 2020 Aug 28;22(8):e19629. doi: 10.2196/19629 (PMC7470238; doi:10.2196/19629)
Supplement: Multimedia Appendix 5 [file jmir_v22i8e19629_app5.docx]

## Multimedia Appendix 5: Categorized Top 250 shared resources (Web site domains)

Total number of occurrences of external references; N = 7,753,841.

| Link Category | Total  Rank | Domain | Occurrences (%) |
| --- | --- | --- | --- |
| **Mainstream or Local News** |  |  | **928467 (11.97)** |
|  | 8 | theguardian.com | 52733 (0.68) |
|  | 11 | nytimes.com | 42735 (0.55) |
|  | 13 | cnn.com | 35494 (0.46) |
|  | 14 | bbc.co.uk | 28286 (0.36) |
|  | 15 | washingtonpost.com | 27316 (0.35) |
|  | 19 | bbc.com | 25853 (0.33) |
|  | 28 | nyti.ms | 17720 (0.23) |
|  | 30 | reuters.com | 17589 (0.23) |
|  | 33 | cnbc.com | 17321 (0.22) |
|  | 35 | bloomberg.com | 16330 (0.21) |
|  | 37 | elpais.com | 15888 (0.20) |
|  | 38 | ouest-france.fr | 14976 (0.19) |
|  | 40 | francetvinfo.fr | 14609 (0.19) |
|  | 41 | scmp.com | 14072 (0.18) |
|  | 43 | reut.rs | 13637 (0.18) |
|  | 46 | forbes.com | 13242 (0.17) |
|  | 48 | nypost.com | 12464 (0.16) |
|  | 49 | businessinsider.com | 12433 (0.16) |
|  | 52 | dailymail.co.uk | 11944 (0.15) |
|  | 53 | sky.com | 11495 (0.15) |
|  | 57 | repubblica.it | 11129 (0.14) |
|  | 59 | wsj.com | 11007 (0.14) |
|  | 61 | independent.co.uk | 10904 (0.14) |
|  | 63 | rt.com | 10222 (0.13) |
|  | 64 | cbc.ca | 10173 (0.13) |
|  | 65 | nbcnews.com | 10021 (0.13) |
|  | 66 | lemonde.fr | 9707 (0.13) |
|  | 67 | thehill.com | 9633 (0.12) |
|  | 68 | indiatimes.com | 9477 (0.12) |
|  | 72 | foxnews.com | 9267 (0.12) |
|  | 73 | npr.org | 9252 (0.12) |
|  | 75 | ft.com | 9059 (0.12) |
|  | 76 | ansa.it | 9008 (0.12) |
|  | 78 | theatlantic.com | 8867 (0.11) |
|  | 81 | usatoday.com | 8415 (0.11) |
|  | 82 | mol.im | 8221 (0.11) |
|  | 89 | apnews.com | 7395 (0.10) |
|  | 90 | telegraph.co.uk | 7356 (0.09) |
|  | 91 | lefigaro.fr | 7208 (0.09) |
|  | 92 | abs-cbn.com | 7123 (0.09) |
|  | 93 | corriere.it | 7091 (0.09) |
|  | 94 | lavanguardia.com | 7089 (0.09) |
|  | 95 | abc.es | 7063 (0.09) |
|  | 96 | elmundo.es | 6962 (0.09) |
|  | 98 | globalnews.ca | 6939 (0.09) |
|  | 99 | latimes.com | 6903 (0.09) |
|  | 101 | leparisien.fr | 6707 (0.09) |
|  | 106 | vox.com | 6497 (0.08) |
|  | 108 | nhk.or.jp | 6495 (0.08) |
|  | 109 | politico.com | 6432 (0.08) |
|  | 112 | efe.com | 6341 (0.08) |
|  | 113 | abc.net.au | 6226 (0.08) |
|  | 118 | india.com | 5916 (0.08) |
|  | 119 | elespanol.com | 5903 (0.08) |
|  | 120 | dw.com | 5778 (0.07) |
|  | 122 | theepochtimes.com | 5646 (0.07) |
|  | 126 | ebcnews.online | 5538 (0.07) |
|  | 127 | jpost.com | 5531 (0.07) |
|  | 130 | googleve.xyz | 5253 (0.07) |
|  | 132 | eldiario.es | 5159 (0.07) |
|  | 134 | sptnkne.ws | 5138 (0.07) |
|  | 137 | huffpost.com | 5038 (0.06) |
|  | 138 | ndtv.com | 5034 (0.06) |
|  | 140 | hindustantimes.com | 4929 (0.06) |
|  | 141 | mirror.co.uk | 4791 (0.06) |
|  | 142 | bbc.in | 4752 (0.06) |
|  | 145 | asianetnews.com | 4687 (0.06) |
|  | 149 | lesechos.fr | 4509 (0.06) |
|  | 156 | europapress.es | 4399 (0.06) |
|  | 158 | time.com | 4372 (0.06) |
|  | 160 | indiatoday.in | 4358 (0.06) |
|  | 161 | actu.fr | 4356 (0.06) |
|  | 162 | ctvnews.ca | 4313 (0.06) |
|  | 163 | aljazeera.com | 4295 (0.06) |
|  | 164 | newsweek.com | 4247 (0.05) |
|  | 165 | ilfattoquotidiano.it | 4225 (0.05) |
|  | 166 | spiegel.de | 4213 (0.05) |
|  | 171 | cgtn.com | 4092 (0.05) |
|  | 176 | amna.gr | 3986 (0.05) |
|  | 177 | globaltimes.cn | 3966 (0.05) |
|  | 178 | cnn.it | 3945 (0.05) |
|  | 180 | 7news.com.au | 3916 (0.05) |
|  | 183 | univision.com | 3817 (0.05) |
|  | 184 | elconfidencial.com | 3775 (0.05) |
|  | 185 | news18.com | 3770 (0.05) |
|  | 186 | faz.net | 3770 (0.05) |
|  | 187 | channelnewsasia.com | 3765 (0.05) |
|  | 188 | francebleu.fr | 3759 (0.05) |
|  | 189 | bfmtv.com | 3735 (0.05) |
|  | 192 | euronews.com | 3711 (0.05) |
|  | 195 | xhne.ws | 3635 (0.05) |
|  | 198 | 20minutes.fr | 3592 (0.05) |
|  | 202 | theglobeandmail.com | 3524 (0.05) |
|  | 203 | alarabiya.net | 3514 (0.05) |
|  | 205 | wired.com | 3499 (0.05) |
|  | 206 | tagesspiegel.de | 3484 (0.04) |
|  | 210 | tagesschau.de | 3408 (0.04) |
|  | 212 | mediaset.it | 3372 (0.04) |
|  | 213 | ilsole24ore.com | 3366 (0.04) |
|  | 215 | lastampa.it | 3348 (0.04) |
|  | 219 | thehindu.com | 3293 (0.04) |
|  | 222 | springnews.co.th | 3279 (0.04) |
|  | 227 | aa.com.tr | 3230 (0.04) |
|  | 228 | arab.news | 3230 (0.04) |
|  | 229 | cadenaser.com | 3226 (0.04) |
|  | 237 | afp.com | 3168 (0.04) |
|  | 238 | cbsnews.com | 3166 (0.04) |
|  | 240 | welt.de | 3100 (0.04) |
|  | 242 | express.co.uk | 3086 (0.04) |
|  | 243 | cbslocal.com | 3084 (0.04) |
|  | 244 | abplive.com | 3078 (0.04) |
|  | 247 | ilmessaggero.it | 3052 (0.04) |
|  | 250 | naftemporiki.gr | 3020 (0.04) |
| **News Blog, Feed,**  **or Niche News** |  |  | **232399 (3.00)** |
|  | 17 | medium.com | 26201 (0.34) |
|  | 42 | zazoom.it | 13926 (0.18) |
|  | 45 | zazoom.info | 13303 (0.17) |
|  | 47 | topicza.com | 12960 (0.17) |
|  | 51 | ecointernet.org | 12028 (0.16) |
|  | 60 | msn.com | 10908 (0.14) |
|  | 80 | flip.it | 8650 (0.11) |
|  | 88 | newsfilter.io | 7628 (0.10) |
|  | 102 | zerohedge.com | 6656 (0.09) |
|  | 103 | poandpo.com | 6629 (0.09) |
|  | 104 | oneindia.com | 6605 (0.09) |
|  | 111 | naciodigital.cat | 6374 (0.08) |
|  | 114 | rawstory.com | 6223 (0.08) |
|  | 121 | statnews.com | 5719 (0.07) |
|  | 123 | breitbart.com | 5623 (0.07) |
|  | 124 | thepigeonexpress.com | 5620 (0.07) |
|  | 125 | theconversation.com | 5541 (0.07) |
|  | 135 | coronavirusnewslive.com | 5069 (0.07) |
|  | 150 | agenparl.eu | 4501 (0.06) |
|  | 167 | infobae.com | 4210 (0.05) |
|  | 168 | theglobalherald.com | 4163 (0.05) |
|  | 182 | latestly.com | 3882 (0.05) |
|  | 190 | tentaran.com | 3727 (0.05) |
|  | 193 | miragenews.com | 3669 (0.05) |
|  | 200 | thegatewaypundit.com | 3574 (0.05) |
|  | 207 | revuedepresse.online | 3464 (0.04) |
|  | 209 | news247worldpressuk.com | 3423 (0.04) |
|  | 211 | thequint.com | 3393 (0.04) |
|  | 214 | theverge.com | 3351 (0.04) |
|  | 216 | presshub.eu | 3348 (0.04) |
|  | 220 | marketwatch.com | 3287 (0.04) |
|  | 224 | 8world.com | 3250 (0.04) |
|  | 235 | konhaber.com | 3172 (0.04) |
|  | 239 | titrespresse.com | 3123 (0.04) |
|  | 241 | rappler.com | 3099 (0.04) |
|  | 246 | rouut.com | 3056 (0.04) |
|  | 248 | socialhot24.com | 3044 (0.04) |
| **Government or Public Health** |  |  | **78786 (1.02)** |
|  | 25 | cdc.gov | 19729 (0.25) |
|  | 27 | who.int | 18298 (0.24) |
|  | 83 | www.gov.uk | 8193 (0.11) |
|  | 84 | nih.gov | 8192 (0.11) |
|  | 85 | europa.eu | 8074 (0.10) |
|  | 148 | canada.ca | 4554 (0.06) |
|  | 152 | medicare4all.org | 4456 (0.06) |
|  | 191 | un.org | 3720 (0.05) |
|  | 201 | www.nhs.uk | 3570 (0.05) |
| **Personal Blog** |  |  | **33571 (0.43)** |
|  | 23 | wordpress.com | 22376 (0.29) |
|  | 55 | wp.me | 11195 (0.14) |
| **Social Network** |  |  | **1406419 (18.14)** |
|  | 1 | twitter.com | 378508 (4.88) |
|  | 2 | youtu.be | 365716 (4.72) |
|  | 3 | instagram.com | 290336 (3.74) |
|  | 5 | youtube.com | 144502 (1.86) |
|  | 6 | facebook.com | 95166 (1.23) |
|  | 7 | linkedin.com | 79787 (1.03) |
|  | 16 | pscp.tv | 26823 (0.35) |
|  | 100 | tiktok.com | 6723 (0.09) |
|  | 115 | twitch.tv | 6198 (0.08) |
|  | 128 | reddit.com | 5453 (0.07) |
|  | 169 | vimeo.com | 4131 (0.05) |
|  | 245 | t.co | 3076 (0.04) |
| **Online Store** |  |  | **40923 (0.53)** |
|  | 20 | amzn.to | 25378 (0.33) |
|  | 58 | amazon.com | 11075 (0.14) |
|  | 151 | ebay.com | 4470 (0.06) |
| **Scientific Resource^a^** |  |  | **22772 (0.29)** |
|  | 116 | nature.com | 6043 (0.08) |
|  | 147 | sciencemag.org | 4615 (0.06) |
|  | 154 | nejm.org | 4405 (0.06) |
|  | 170 | medrxiv.org | 4123 (0.05) |
|  | 199 | jhu.edu | 3586 (0.05) |
| **URL Shortener^a^** |  |  | **106588 (1.37)** |
|  | 10 | tinyurl.com | 44768 (0.58) |
|  | 24 | trib.al | 21409 (0.28) |
|  | 86 | cutt.ly | 8034 (0.10) |
|  | 87 | bit.ly | 7743 (0.10) |
|  | 131 | spr.ly | 5223 (0.07) |
|  | 139 | zpr.io | 4970 (0.06) |
|  | 153 | glblctzn.me | 4444 (0.06) |
|  | 208 | tiny.cc | 3433 (0.04) |
|  | 217 | ow.ly | 3320 (0.04) |
|  | 225 | shorturl.at | 3244 (0.04) |
| **Other** |  |  | **746613 (9.63)** |
|  | 4 | paper.li | 204077 (2.63) |
|  | 9 | google.com | 47184 (0.61) |
|  | 12 | chng.it | 41316 (0.53) |
|  | 18 | fiverr.com | 25905 (0.33) |
|  | 21 | ift.tt | 23304 (0.30) |
|  | 22 | avaaz.org | 22938 (0.30) |
|  | 26 | arcgis.com | 18604 (0.24) |
|  | 29 | worldometers.info | 17670 (0.23) |
|  | 31 | yahoo.com | 17588 (0.23) |
|  | 32 | apple.news | 17584 (0.23) |
|  | 34 | openstream.co | 16442 (0.21) |
|  | 36 | goo.gl | 16310 (0.21) |
|  | 39 | joinzoe.com | 14827 (0.19) |
|  | 44 | shoutcast.com | 13635 (0.18) |
|  | 50 | dy.si | 12343 (0.16) |
|  | 54 | vuhere.com | 11445 (0.15) |
|  | 56 | us-central1-thaishare-prod.cloudfunctions.net | 11191 (0.14) |
|  | 62 | spotify.com | 10674 (0.14) |
|  | 69 | threadreaderapp.com | 9361 (0.12) |
|  | 70 | soundcloud.com | 9291 (0.12) |
|  | 71 | apple.com | 9279 (0.12) |
|  | 74 | gofundme.com | 9204 (0.12) |
|  | 77 | change.org | 8918 (0.12) |
|  | 79 | covidworld.info | 8719 (0.11) |
|  | 97 | zoom.us | 6954 (0.09) |
|  | 105 | weforum.org | 6528 (0.08) |
|  | 107 | mailchi.mp | 6496 (0.08) |
|  | 110 | yahoo.co.jp | 6374 (0.08) |
|  | 117 | t.me | 5973 (0.08) |
|  | 129 | wikipedia.org | 5436 (0.07) |
|  | 133 | thelancet.com | 5153 (0.07) |
|  | 136 | lasopa.com | 5044 (0.07) |
|  | 143 | spreaker.com | 4698 (0.06) |
|  | 144 | 38degrees.org.uk | 4690 (0.06) |
|  | 146 | bing.com | 4632 (0.06) |
|  | 155 | metallicradio.com | 4400 (0.06) |
|  | 157 | actionnetwork.org | 4374 (0.06) |
|  | 159 | shar.es | 4365 (0.06) |
|  | 172 | staythefuckhome.com | 4077 (0.05) |
|  | 173 | smh.re | 4022 (0.05) |
|  | 174 | allexpressnews.com | 4013 (0.05) |
|  | 175 | covidstat.app | 4012 (0.05) |
|  | 179 | zcu.io | 3937 (0.05) |
|  | 181 | paperbirds.me | 3908 (0.05) |
|  | 194 | situazionecoronavirus.it | 3666 (0.05) |
|  | 196 | ncr-iran.org | 3631 (0.05) |
|  | 197 | go.com | 3608 (0.05) |
|  | 204 | sumof.us | 3513 (0.05) |
|  | 218 | pwastart.com | 3313 (0.04) |
|  | 221 | shr.lc | 3280 (0.04) |
|  | 223 | github.com | 3277 (0.04) |
|  | 226 | mtr.cool | 3230 (0.04) |
|  | 230 | okt.to | 3217 (0.04) |
|  | 231 | rviv.ly | 3209 (0.04) |
|  | 232 | anchor.fm | 3199 (0.04) |
|  | 233 | radio.com | 3191 (0.04) |
|  | 234 | pst.cr | 3180 (0.04) |
|  | 236 | ampproject.org | 3169 (0.04) |
|  | 249 | mapsofworld.com | 3035 (0.04) |

^a^ Link category as extension to the list given in Chew and Eysenbach [31], Table 3.
